# Supplementary material for: Cas9-mediated knockout of Ndrg2 enhances the regenerative potential of dendritic cells for wound healing
Source: Nat Commun. 2023 Aug 7;14:4729. doi: 10.1038/s41467-023-40519-z (PMC10406832; doi:10.1038/s41467-023-40519-z)
Supplement: Supplementary file 1 — Supplementary Information [file 41467_2023_40519_MOESM1_ESM.pdf]

Table S1: Top differentially expressed genes in clusters in Figure 1b. Differentially expressed genes were determined using a Wilcoxon Rank Sum test and P-values were adjusted using the Benjamini-Hochberg procedure as part of the Seurat package.

Gene	Cluster	Average log2 fold-change	P value	Adjusted p value
Ccl5	0	1.23795363	0	0
Ccr7	0	1.20732704	0	0
Ccl22	0	1.18237658	0	0
Il4i1	0	1.07817967	0	0
Cacnb3	0	1.07180507	0	0
Pkib	0	1.06838631	0	0
Cd83	0	0.97497187	0	0
AW112010	0	0.94416737	0	0
Fscn1	0	0.93953244	0	0
Tmem123	0	0.89994552	0	0
Mmp12	1	0.95990141	0	0
Lyz2	1	0.87841023	0	0
Fn1	1	0.85910715	0	0
Ccr2	1	0.8473583	0	0
Cybb	1	0.83979473	0	0
Ly6e	1	0.80904562	0	0
Spp1	1	0.78345409	0	0
Axl	1	0.77448159	0	0
Mgl2	1	0.76409805	0	0
Mki67	1	0.75081622	0	0
Stmn11	2	0.50569475	4.29E-304	7.01E-300
Tagln21	2	0.25808873	5.60E-274	9.15E-270
Atp5g11	2	0.27311474	3.16E-273	5.16E-269
Ifitm11	2	0.48557737	5.96E-266	9.74E-262
Eif5a1	2	0.34342179	1.26E-262	2.05E-258
Mif1	2	0.38169305	1.23E-250	2.02E-246
S100a8	2	0.31298475	8.45E-231	1.38E-226
Hmgb21	2	0.4272201	6.64E-222	1.08E-217
Prdx51	2	0.31630257	1.06E-220	1.73E-216
Ifitm31	2	0.44180202	1.02E-219	1.67E-215
S100a81	3	1.3232708	0	0
Lst1	3	1.06134597	0	0
Slpi	3	1.05139218	0	0
F630028O10Rik	3	0.94078026	0	0
Ier31	3	0.93976792	0	0
Lcn2	3	0.93265877	0	0
Wfdc211	3	0.92906298	0	0
S100a91	3	0.92668224	0	0
Chil31	3	0.88205647	0	0
Gsr	3	0.8608867	0	0
Msr11	4	0.38103331	5.20E-124	8.49E-120
Gpnmb1	4	0.43564069	1.27E-110	2.08E-106
C1qc1	4	0.43156007	1.29E-110	2.11E-106
Mmp141	4	0.28647519	2.42E-97	3.95E-93
Mki671	4	0.37735999	3.12E-97	5.10E-93
Ccl221	4	0.44385663	1.33E-94	2.17E-90
Dab21	4	0.32914391	3.02E-94	4.93E-90
C1qa1	4	0.44602833	1.01E-93	1.64E-89
Ccl62	4	0.43199968	2.03E-93	3.31E-89
Ccl21	4	0.3398543	2.72E-83	4.44E-79
Tcirg11	5	0.26955729	3.69E-48	6.02E-44
Lars2	5	0.48537661	2.49E-37	4.07E-33
Gm269171	5	0.42220086	1.70E-08	0.00027745

**Itgae**	6	1.18020713	0	0
**Tcea3**	6	0.9833812	0	0
**Kmo**	6	0.75531085	0	0
**Tespa1**	6	0.67434064	0	0
**Kctd14**	6	0.67423954	0	0
**Havcr2**	6	0.63435135	0	0
**Klrb1b**	6	0.63037814	0	0
**Clec9a**	6	0.60050402	0	0
**Dmpk**	6	0.59482933	0	0
**Rtn1**	6	0.5290375	0	0
**Prss34**	7	1.44942023	0	0
**Mcpt8**	7	1.41800277	0	0
**Cpa3**	7	1.30794931	0	0
**Ctla2a**	7	1.27443479	0	0
**Cyp11a1**	7	1.17349468	0	0
**Cd200r3**	7	1.02752842	0	0
**Gata2**	7	0.99394836	0	0
**Fcer1a**	7	0.87978124	0	0
**Ms4a2**	7	0.87253855	0	0
**Ptprcap**	7	0.87038202	0	0

Table S2: Spacer sequences of Ndrg2 sgRNA, negative control sgRNAs, primer sequences

NDRG2 sgRNA	sgRNA Space Sequence	sgRNA targeting Exon/intron	Manufacturer
NDRG2.1.AC	GTAAAAGTGACCGAGCCATA	Exon4	IDT
NDRG2.1.AA	CCAGCCACTGTTTCGGTTCG	Exon5	IDT
NDRG2.1.AE	ACAATAATTGGAGTTGGTGT	Exon7	IDT
NDRG2_1	ATGTTCAGAGCATGGGACCG	Exon2	Synthego
NDRG2_2	GCAGTCTCGGGTGTTTGTCC	Exon2	Synthego
NDRG2_3	CTCCTGAAGTTCTGCCATGG	Intron2	Synthego
**Negative control sgRNA**			
NC1	CGTTAATCGCGTATAATACG		IDT
NC2	CATATTGCGCGTATAGTCGC		IDT
NC3	GGCGCGTATAGTCGCGCGTA		IDT
**Primer for PCR and sequencing**	**Primer sequence**		
NDRG2.1.AC_For	TCTGCATCCCTCCCATTTATCCTCT		
NDRG2.1.AC_Rev	CAGAACCCACCCTCTTGCTCTGATG		
NDRG2.1.AA_For	CCATGATGTAGGCCTCAACTGTAAGAC		
NDRG2.1.AA_Rev	TCTTCTCTGGAGCAACCTCCGCA		
NDRG2.1.AE_For	CAGAGAGCCGAGAATTGATAAACTC		
NDRG2.1.AE_Rev	GCAGGAACTCGGACCATTCCTGCAA		
NDRG2-3mix-F	CTGGTGTTGCTCTGCACCTTGCATTG		
NDRG2-3mix-R	TGGTTACCTGTCCCTGGTCCAGGAG		

Table S3: 14 sites with detected indels, located 200 base pairs up- and down-stream of the sites predicted by CasOFFinder

Genomic Position	REF	ALT	AffectedGene	VEP_Consequence	VariantClass
chr3:113572900	ATG	A	Amy1	intron_variant	deletion
chr3:113572900	ATG	A	Amy1	intron_variant	deletion
chr3:113572900	ATG	A	Amy1	intron_variant	deletion
chr3:113572900	ATG	A	Amy1	intron_variant	deletion
chr4:31726093	T	TG		intergenic_variant	insertion
chr9:89597121	AT	A	Minar1	intron_variant	deletion
chr9:89597121	AT	A	Minar1	intron_variant&NMD_transcript_variant	deletion
chr15:32342480	GA	G	Sema5a	intron_variant	deletion
chr15:32342480	GA	G	Sema5a	intron_variant&non_coding_transcript_variant	deletion
chr15:32342480	GA	G	Sema5a	intron_variant&non_coding_transcript_variant	deletion
chr15:32342480	GA	G	Sema5a	intron_variant&non_coding_transcript_variant	deletion
chr15:32342480	GA	G	Sema5a	intron_variant&non_coding_transcript_variant	deletion
chr15:32342480	GA	G	Sema5a	intron_variant&non_coding_transcript_variant	deletion
chr15:32342480	GA	G	Sema5a	intron_variant&non_coding_transcript_variant	deletion
chrX:8236875	GT	G	Ftsj1	downstream_gene_variant	deletion
chrX:8236875	GT	G	Ftsj1	downstream_gene_variant	deletion
chrX:8236875	GT	G	Ftsj1	downstream_gene_variant	deletion
chrX:48305938	AT	A	Gm7212	upstream_gene_variant	deletion
chrX:48305938	AT	A	Gm22612	upstream_gene_variant	deletion
chrX:52002943	AACAC	A		intergenic_variant	deletion
chrX:63795577	TCA	T		regulatory_region_variant	deletion
chrX:63795577	TCA	T		intergenic_variant	deletion
chrX:73054780	ATC	A	Gm6858	downstream_gene_variant	deletion
chrX:73054780	ATC	A		regulatory_region_variant	deletion
chrX:96613198	TTTG	T		intergenic_variant	deletion
chrX:116833769	GTA	G		intergenic_variant	deletion
chrX:126131620	TA	T	Gm14957	intron_variant&non_coding_transcript_variant	deletion
chrX:136367996	G	GT	Gm14998	downstream_gene_variant	insertion
chrX:136367996	G	GT	Gm14993	upstream_gene_variant	insertion

Table S4: Top differentially expressed genes in clusters in Figure 3b. Differentially expressed genes were determined using a Wilcoxon Rank Sum test and P-values were adjusted using the Benjamini-Hochberg procedure as part of the Seurat package.

Gene	Cluster	Average log2 fold-change	P value	Adjusted p value
**Ccr7**	0	1.308721	0	0
**Ccl5**	0	1.282455	0	0
**Ccl22**	0	1.234959	0	0
**Fscn1**	0	1.201093	0	0
**Cacnb3**	0	1.163427	0	0
**Il4i1**	0	1.126532	0	0
**Cd83**	0	1.0868	0	0
**Pkib**	0	1.036909	0	0
**Cytip**	0	1.022912	0	0
**AW112010**	0	0.970083	0	0
**Fn1**	1	1.474758	0	0
**Mmp12**	1	1.444099	0	0
**Cd36**	1	1.259814	0	0
**Wfdc17**	1	1.214793	0	0
**Pf4**	1	1.037544	0	0
**Ctsl**	1	1.012257	0	0
**Cybb**	1	1.006228	0	0
**Arg1**	1	0.991243	0	0
**Gpnmb**	1	0.969484	0	0
**Ccl9**	1	0.951666	0	0
**Chil3**	2	1.300463	0	0
**S100a8**	2	0.894305	0	0
**Ly6e**	2	0.84073	0	0
**Ier3**	2	0.737295	0	0
**Lcn2**	2	0.730652	0	0
**Emb**	2	0.688769	0	0
**Clec12a**	2	0.610218	0	0
**Wfdc21**	2	0.608925	0	0
**Cfp1**	2	0.601045	0	0
**Ly6c2**	2	0.60065	0	0
**Nr4a31**	3	0.892812	0	0
**Ccl221**	3	0.817902	0	0
**Tnfrsf91**	3	0.816643	0	0
**Ccl51**	3	0.754444	0	0
**Ccr71**	3	0.739118	0	0
**Ccl17**	3	0.729048	0	0
**Pkib1**	3	0.715226	0	0
**Tmem1231**	3	0.680082	0	0
**Il4i11**	3	0.679843	0	0
**Aldh1a2**	3	0.672576	0	0
**Eif5a1**	4	0.440302	0	0
**Tagln2**	4	0.342958	0	0
**Taldo1**	4	0.278537	0	0
**Cotl11**	4	0.272757	8.12631879731532E-295	1.41365441798097E-290
**Atp5g1**	4	0.310672	3.15884662164848E-254	5.49512958301969E-250
**Ifitm11**	4	0.750444	4.76018946093358E-183	8.28082558624006E-179
**Ran**	4	0.302428	7.27096748090682E-177	1.26485750297855E-172
**Prdx52**	4	0.26188	4.52997514999536E-159	7.88034477093194E-155
**Rac21**	4	0.338964	1.30407291429381E-156	2.26856524170552E-152
**Mif1**	4	0.383002	2.00049747709312E-153	3.48006541115118E-149

**Stmn1**	4	0.628506	1.64681612760145E-144	2.86480133557548E-140
**Stmn11**	5	1.045564	0	0
**Pclaf1**	5	1.011041	0	0
**Top2a**	5	0.915394	0	0
**Birc51**	5	0.910138	0	0
**Ube2c1**	5	0.897679	0	0
**Mki67**	5	0.864373	0	0
**Mmp121**	5	0.86306	0	0
**Cks1b1**	5	0.749258	0	0
**Smc2**	5	0.727897	0	0
**Cdca3**	5	0.724758	0	0
**Nr4a32**	6	0.356689	4.50195053087782E-234	7.83159314351506E-230
**Ccl222**	6	0.589671	2.47281260542309E-204	4.30170480839401E-200
**Tnfrsf92**	6	0.321028	7.06791022017134E-168	1.22953366190101E-163
**Ccl52**	6	0.567839	1.87709259737664E-158	3.2653902823964E-154
**Net12**	6	0.264507	1.37860361093843E-146	2.3982188415885E-142
**Tmem1232**	6	0.398612	3.48916423585996E-134	6.06975010470199E-130
**Ccr72**	6	0.448361	2.0644668873581E-129	3.59134659724814E-125
**Etv32**	6	0.313902	3.40432617570419E-128	5.922165815255E-124
**AW1120102**	6	0.401676	3.07158679863772E-123	5.34333239491018E-119
**Il4i12**	6	0.300869	6.20757055823537E-117	1.07986897431062E-112
**S100a81**	7	1.439091	0	0
**Lst11**	7	1.412298	0	0
**Slpi1**	7	1.308631	0	0
**Ier31**	7	1.13548	0	0
**Mmp91**	7	1.12282	0	0
**Id11**	7	1.035611	0	0
**F630028O10Rik1**	7	1.01898	0	0
**Ier21**	7	0.988031	0	0
**Wfdc211**	7	0.978093	0	0
**Gsr1**	7	0.976737	0	0
**Chil32**	8	1.264465	0	0
**S100a92**	8	1.254139	0	0
**S100a82**	8	1.223117	0	0
**Ly6c21**	8	1.171467	0	0
**Hmgb22**	8	1.070297	0	0
**Stmn12**	8	1.058509	0	0
**Birc52**	8	1.04588	0	0
**Top2a1**	8	1.044832	0	0
**Mki671**	8	1.028179	0	0
**Pclaf2**	8	1.008413	0	0
**Plet12**	9	1.244277	0	0
**Asb2**	9	0.778888	0	0
**Itgae**	9	0.534675	0	0
**Kmo**	9	0.468977	0	0
**Kctd14**	9	0.344123	0	0
**Scimp**	9	0.827456	1.3604561125443E-286	2.36664945338206E-282
**Dmpk**	9	0.268656	6.40704164756463E-283	1.11456896501034E-278
**Cldn1**	9	0.305402	6.71707698791199E-266	1.16850271281717E-261
**Trp53i11**	9	0.465016	3.10907041560873E-260	5.40853889499295E-256
**Itgb7**	9	0.671556	2.6595400970498E-254	4.62653595282784E-250
**Tspo1**	10	0.355188	2.6224375387713E-105	4.56199234244655E-101
**AW1120103**	10	0.77974	5.16531943015335E-103	8.98558968069476E-99
**Bcl2a1b1**	10	0.335699	3.87450972781613E-102	6.74009712250895E-98
**Eif1**	10	0.258591	1.82245796780901E-95	3.17034788080056E-91
**Ccl53**	10	0.940423	9.12170849171685E-94	1.58681240921906E-89

**Psmb81**	10	0.402373	2.50850659998633E-92	4.36379808133622E-88
**Txndc172**	10	0.423891	3.72860878246512E-92	6.48628783797633E-88
**Pkib3**	10	0.683942	3.90394446698253E-84	6.79130179476281E-80
**Pfdn51**	10	0.260391	6.78175200949878E-77	1.17975357957241E-72
**Cox7a2l2**	10	0.32868	5.83019739923882E-75	1.01422113957158E-70
**Prss34**	11	1.131666	0	0
**Mcpt8**	11	1.106258	0	0
**Ctla2a**	11	1.094147	0	0
**Cpa3**	11	0.989774	0	0
**Gata2**	11	0.74884	0	0
**Ptprcap**	11	0.663903	0	0
**Ms4a2**	11	0.64438	0	0
**Itga2b**	11	0.544366	0	0
**Hdc**	11	0.535634	0	0
**F2r**	11	0.42335	0	0

Table S5: Top differentially expressed genes in fibroblasts of wounds treated with control dendritic cells (Wound_Controls) or Ndrg2KO dendritic cells (Wound_DC _Ndrg2KO. Differentially expressed genes were determined using a Wilcoxon Rank Sum test and P-values were adjusted using the Benjamini-Hochberg procedure as part of the Seurat package.

Gene	Group	Average log2 fold-change	P value	Adjusted p value
**Lars2**	Wound_Controls	1.144736238	4.22300941231472E-86	7.86028741914139E-82
**Ace**	Wound_Controls	0.838384191	4.08060455648684E-36	7.59522926098896E-32
**Zbtb16**	Wound_Controls	0.431649009	3.1180856879325E-28	5.80369289094877E-24
**Fndc1**	Wound_Controls	0.625723437	4.80225426192056E-27	8.93843585771274E-23
**Ctsk**	Wound_Controls	0.794532256	6.67497665953944E-27	1.24241340564008E-22
**Tsc22d3**	Wound_Controls	0.412523057	3.32421066516863E-25	6.18735331107837E-21
**Fam102b**	Wound_Controls	0.430812328	1.13707650075107E-24	2.11644049084798E-20
**Slc38a2**	Wound_Controls	0.472028557	1.35212208146683E-24	2.51670483023421E-20
**Epas1**	Wound_Controls	0.402901436	6.12651616863804E-20	1.1403284544686E-15
**Agap1**	Wound_Controls	0.261405795	1.80869934270163E-19	3.36653208657054E-15
**Islr2**	Wound_Controls	0.288040664	8.29110825166609E-19	1.54322397888261E-14
**Gxylt2**	Wound_Controls	0.38323028	1.0015450498015E-18	1.86417580119553E-14
**Aebp1**	Wound_Controls	0.366849168	4.37992664594992E-18	8.15235746610659E-14
**Galnt15**	Wound_Controls	0.470440975	1.99161111240618E-17	3.70698576352163E-13
**Dkk3**	Wound_Controls	0.266397246	4.5394588166009E-17	8.44929469533925E-13
**Gpnmb**	Wound_Controls	0.452357996	5.80957564747695E-17	1.08133631526489E-12
**Ddr2**	Wound_Controls	0.423679624	8.19841269845012E-17	1.52597055556252E-12
**Celf2**	Wound_Controls	0.368291177	1.0967876275645E-16	2.0414508111858E-12
**Fmo2**	Wound_Controls	0.277862938	2.89544291248182E-16	5.38928789300241E-12
**Pamr1**	Wound_Controls	0.5278337	3.19435512724027E-16	5.94565319833232E-12
**Itga11**	Wound_Controls	0.269282253	4.26502532428793E-16	7.93849163609713E-12
**Wsb1**	Wound_Controls	0.475781753	4.63456371612558E-16	8.62631344482453E-12
**Dpt**	Wound_Controls	0.36908845	9.561785407073E-16	1.7797351178185E-11
**Atf4**	Wound_Controls	0.330668121	1.08084747870001E-15	2.01178141210433E-11
**Pltp**	Wound_Controls	0.384779936	1.57440527790235E-15	2.93044054375965E-11
**Mmp2**	Wound_Controls	0.355407159	2.76161580058804E-15	5.14019548963452E-11
**Lmo4**	Wound_Controls	0.324496678	3.85405137050589E-15	7.17354581592262E-11
**Med13l**	Wound_Controls	0.276667454	1.03493057320249E-14	1.9263162759018E-10
**Hsd11b1**	Wound_Controls	0.3160274	1.42646184452771E-14	2.65507343121943E-10
**Islr**	Wound_Controls	0.427814046	1.98355998554667E-14	3.69200020109801E-10
**Igf1r**	Wound_Controls	0.26512145	3.07548159047999E-14	5.72439388436041E-10
**Lamb1**	Wound_Controls	0.281257277	4.48662382145972E-14	8.35095291888297E-10
**C1s1**	Wound_Controls	0.377507761	4.5824100023719E-14	8.52923973741482E-10
**Anpep**	Wound_Controls	0.359595069	7.30913475548639E-14	1.36044925203868E-09
**Xdh**	Wound_Controls	0.416024008	8.48084308467572E-14	1.57853932335069E-09
**Scpep1**	Wound_Controls	0.270813848	9.51357912123029E-14	1.77076248183459E-09
**Timp2**	Wound_Controls	0.402395297	1.04922957861272E-13	1.95293101467185E-09
**Ahnak2**	Wound_Controls	0.37261968	1.15323205702314E-13	2.14651082773717E-09
**C4b**	Wound_Controls	0.542638916	1.57261230162011E-13	2.92710327700552E-09
**Itgbl1**	Wound_Controls	0.42318835	3.98196407343659E-13	7.41162972988753E-09
**Ctla2a**	Wound_Controls	1.045701015	4.07667125926801E-13	7.58790821487554E-09
**Kdm7a**	Wound_Controls	0.264279283	4.16359099021451E-13	7.74969191008627E-09
**Tgfbi**	Wound_Controls	0.295280535	7.63127895447245E-13	1.42040995179596E-08
**Nbl1**	Wound_Controls	0.36867983	1.43332562290519E-12	2.66784898191342E-08
**Dapk1**	Wound_Controls	0.359443358	2.12305797774833E-12	3.95164781398296E-08
**Hmox1**	Wound_Controls	0.597131911	3.09064726104485E-12	5.75262174698278E-08
**Phldb1**	Wound_Controls	0.282876784	4.27176297743744E-12	7.95103242990432E-08
**Fgfr1**	Wound_Controls	0.298112662	4.5503540579669E-12	8.46957400809379E-08
**Mmp14**	Wound_Controls	0.34492723	4.72400171780366E-12	8.79278439734795E-08
**Tgfbr3**	Wound_Controls	0.325883659	5.8226893037105E-12	1.08377716009964E-07
**Ppib**	Wound_DC_Ndrg2KO	0.639459582	3.36015173568933E-73	6.25425042563855E-69

**Ngfr**	Wound_DC_Ndrg2KO	0.68293295	6.89383526027274E-71	1.28314955699457E-66
**Plod2**	Wound_DC_Ndrg2KO	0.965680526	1.25617164958547E-69	2.33811229137344E-65
**Ptpn5**	Wound_DC_Ndrg2KO	0.525216947	1.17633162169512E-67	2.18950604746112E-63
**Selenow**	Wound_DC_Ndrg2KO	0.599130026	6.07089803600329E-67	1.12997625144129E-62
**Ssr4**	Wound_DC_Ndrg2KO	0.740112712	3.02572513380371E-65	5.63178219154884E-61
**Ctsh**	Wound_DC_Ndrg2KO	0.736249563	3.65573157568818E-60	6.8044131818284E-56
**Lgals1**	Wound_DC_Ndrg2KO	0.920545677	8.83436376919921E-60	1.64434012836105E-55
**Serf2**	Wound_DC_Ndrg2KO	0.558681077	2.59204630496582E-58	4.82457578743288E-54
**Gapdh**	Wound_DC_Ndrg2KO	0.732763788	7.91583176069135E-58	1.47337376561748E-53
**Cd320**	Wound_DC_Ndrg2KO	0.566656444	8.30037014288694E-56	1.54494789469555E-51
**Sec61g**	Wound_DC_Ndrg2KO	0.615779361	2.06202879921485E-54	3.8380542039786E-50
**S100a6**	Wound_DC_Ndrg2KO	0.708755589	2.37854661412516E-51	4.42718881287117E-47
**Ndufa13**	Wound_DC_Ndrg2KO	0.475182634	5.99781349423709E-51	1.11637302568235E-46
**Sec61b**	Wound_DC_Ndrg2KO	0.651050055	1.63466365619422E-50	3.0425994632743E-46
**Pgr**	Wound_DC_Ndrg2KO	0.498407683	3.37128959243517E-49	6.27498131839958E-45
**S100a16**	Wound_DC_Ndrg2KO	0.678284412	7.59375197335966E-49	1.41342505480143E-44
**Elob**	Wound_DC_Ndrg2KO	0.520206299	8.0818092207497E-49	1.50426715025814E-44
**Greb1**	Wound_DC_Ndrg2KO	0.336601722	1.66803437877617E-48	3.10471238921608E-44
**Ifi27l2a**	Wound_DC_Ndrg2KO	1.204712828	2.4975766093499E-47	4.64873934298297E-43
**Prdx5**	Wound_DC_Ndrg2KO	0.613516826	9.51430434725287E-47	1.77089746815418E-42
**Maged2**	Wound_DC_Ndrg2KO	0.566136838	1.17084959692316E-45	2.17930235475308E-41
**Selenom**	Wound_DC_Ndrg2KO	0.562940228	1.31208248006291E-45	2.44217912014109E-41
**Atp5j2**	Wound_DC_Ndrg2KO	0.49027922	2.38237095944759E-44	4.4343070668198E-40
**Park7**	Wound_DC_Ndrg2KO	0.49651136	3.38392008136174E-43	6.29849044743861E-39
**Spon2**	Wound_DC_Ndrg2KO	0.690185551	3.42744046392104E-43	6.37949493549623E-39
**Lamtor2**	Wound_DC_Ndrg2KO	0.439082667	5.01221907638172E-42	9.3292433668693E-38
**Serpinb6a**	Wound_DC_Ndrg2KO	0.771473346	1.30926201129495E-41	2.43692938162329E-37
**Atp5e**	Wound_DC_Ndrg2KO	0.395822978	1.73770911953678E-41	3.2343979841938E-37
**Atp5g1**	Wound_DC_Ndrg2KO	0.546857267	7.54829853118917E-41	1.40496480561024E-36
**Prdx4**	Wound_DC_Ndrg2KO	0.592111937	1.77250016745338E-40	3.29915456168098E-36
**Swi5**	Wound_DC_Ndrg2KO	0.444064008	7.04611133594391E-39	1.31149270295924E-34
**Gng5**	Wound_DC_Ndrg2KO	0.404375339	1.5169013387322E-38	2.82340846178224E-34
**Prdx2**	Wound_DC_Ndrg2KO	0.449974686	4.24462731426093E-38	7.90052482003387E-34
**Cox6b1**	Wound_DC_Ndrg2KO	0.418827052	9.94734787451128E-38	1.85149985988279E-33
**Edf1**	Wound_DC_Ndrg2KO	0.45381332	1.17998443238443E-37	2.19630502399714E-33
**Ndufa4**	Wound_DC_Ndrg2KO	0.510918636	1.31081202720804E-37	2.43981442624232E-33
**Slc25a4**	Wound_DC_Ndrg2KO	0.415363716	1.5210117921487E-37	2.83105924872638E-33
**Cox5b**	Wound_DC_Ndrg2KO	0.463461228	2.64012334935084E-37	4.91406159014672E-33
**Calm1**	Wound_DC_Ndrg2KO	0.487467797	8.12958329209149E-37	1.51315933815699E-32
**Arpc1b**	Wound_DC_Ndrg2KO	0.429124958	8.38053468455512E-37	1.55986892083624E-32
**Csrp2**	Wound_DC_Ndrg2KO	0.764468521	5.75038455802496E-36	1.07031907778519E-31
**Uqcrq**	Wound_DC_Ndrg2KO	0.463083522	2.32299695018587E-35	4.32379422338097E-31
**Uqcr11**	Wound_DC_Ndrg2KO	0.416650021	3.59943836597115E-35	6.69963463058211E-31
**Pcolce**	Wound_DC_Ndrg2KO	0.502285158	3.91056688818356E-35	7.27873814897605E-31
**S100a13**	Wound_DC_Ndrg2KO	0.473149122	5.46480522300504E-35	1.01716419615793E-30
**Ppia**	Wound_DC_Ndrg2KO	0.460854373	1.68623348766193E-34	3.13858639058516E-30
**Il4ra**	Wound_DC_Ndrg2KO	0.473119159	3.59516466311494E-34	6.69167998745583E-30
**Ndufs5**	Wound_DC_Ndrg2KO	0.409011786	5.22540400375285E-34	9.72604447218518E-30
**Ost4**	Wound_DC_Ndrg2KO	0.436801228	7.4973303405309E-34	1.39547809628302E-29

Table S6: Top differentially expressed genes in myeloid cells of wounds treated with control dendritic cells (Wound_Controls) or Ndrg2KO dendritic cells (Wound_DC _Ndrg2KO. Differentially expressed genes were determined using a Wilcoxon Rank Sum test and P-values were adjusted using the Benjamini-Hochberg procedure as part of the Seurat package.

Gene	Group	Average log2 fold-change	P value	Adjusted p value
**Fkbp5**	Wound_Controls	0.59021312	6.88E-103	1.28E-98
**Man2a1**	Wound_Controls	0.64439123	5.59E-76	1.04E-71
**Wnk1**	Wound_Controls	0.50658489	4.58E-54	8.52E-50
**Rassf2**	Wound_Controls	0.43065913	1.69E-44	3.14E-40
**Mmp19**	Wound_Controls	0.87442687	9.25E-44	1.72E-39
**Cxcr4**	Wound_Controls	0.54937287	3.74E-40	6.95E-36
**Lgmn**	Wound_Controls	0.59626957	1.70E-39	3.17E-35
**Rbm47**	Wound_Controls	0.41159955	5.72E-37	1.06E-32
**Cebpa**	Wound_Controls	0.45236152	9.36E-37	1.74E-32
**Serinc3**	Wound_Controls	0.55275104	1.21E-36	2.25E-32
**Ucp2**	Wound_Controls	0.39721775	1.99E-36	3.70E-32
**Ctsb**	Wound_Controls	0.57369601	5.95E-36	1.11E-31
**Tsc22d3**	Wound_Controls	0.51697428	3.93E-35	7.32E-31
**Atf4**	Wound_Controls	0.45022067	6.55E-35	1.22E-30
**Laptm5**	Wound_Controls	0.32223537	1.56E-32	2.91E-28
**BC005537**	Wound_Controls	0.38040571	3.93E-32	7.32E-28
**Clec4a1**	Wound_Controls	0.44956331	3.57E-31	6.64E-27
**Slc38a2**	Wound_Controls	0.3596702	1.80E-30	3.35E-26
**Arpc4**	Wound_Controls	0.37951018	1.91E-30	3.56E-26
**Mertk**	Wound_Controls	0.35310143	6.91E-30	1.29E-25
**Canx**	Wound_Controls	0.29369726	5.36E-29	9.97E-25
**Ctss**	Wound_Controls	0.3958103	2.63E-28	4.89E-24
**Atp1a1**	Wound_Controls	0.31187347	1.59E-27	2.95E-23
**Il6ra**	Wound_Controls	0.37039879	2.44E-27	4.55E-23
**Abl2**	Wound_Controls	0.36687521	7.14E-27	1.33E-22
**Mrc1**	Wound_Controls	0.57952794	1.03E-26	1.92E-22
**Ptp4a2**	Wound_Controls	0.28333709	1.78E-26	3.31E-22
**Grn**	Wound_Controls	0.41330258	1.97E-26	3.67E-22
**Tns1**	Wound_Controls	0.46358041	2.19E-26	4.08E-22
**Tob2**	Wound_Controls	0.3381922	7.04E-26	1.31E-21
**Spop**	Wound_Controls	0.2943754	7.62E-26	1.42E-21
**Cln8**	Wound_Controls	0.33758474	3.15E-25	5.86E-21
**Rab7b**	Wound_Controls	0.44809511	3.57E-25	6.64E-21
**Ms4a6d**	Wound_Controls	0.33306331	4.03E-25	7.49E-21
**Zmiz1**	Wound_Controls	0.3218535	2.61E-24	4.86E-20
**Inpp5d**	Wound_Controls	0.28300613	3.39E-24	6.31E-20
**Glul**	Wound_Controls	0.50257497	8.37E-24	1.56E-19
**Scpep1**	Wound_Controls	0.28811068	3.04E-23	5.65E-19
**Itm2c**	Wound_Controls	0.31616565	6.31E-23	1.17E-18
**Ahnak**	Wound_Controls	0.30807452	1.06E-22	1.98E-18
**Tet3**	Wound_Controls	0.28858284	1.73E-21	3.23E-17
**Sik1**	Wound_Controls	0.33654004	2.20E-21	4.10E-17
**Ubl3**	Wound_Controls	0.25974547	2.21E-21	4.11E-17
**Csnk1a1**	Wound_Controls	0.26122428	2.73E-21	5.07E-17
**Rtn4**	Wound_Controls	0.25795823	4.13E-21	7.69E-17
**Ddx3x**	Wound_Controls	0.32991191	5.39E-21	1.00E-16
**Frmd4b**	Wound_Controls	0.38570991	5.97E-21	1.11E-16
**Clec4a3**	Wound_Controls	0.28414711	9.71E-21	1.81E-16
**Atp6v1b2**	Wound_Controls	0.31487347	1.19E-20	2.21E-16
**Taok1**	Wound_Controls	0.27669037	2.11E-20	3.93E-16

**Fcer1g**	Wound_DC_Ndrg2KO	0.51323384	1.11E-78	2.06E-74
**Cxcl2**	Wound_DC_Ndrg2KO	1.23955214	1.81E-77	3.37E-73
**Ubb**	Wound_DC_Ndrg2KO	0.63218136	1.43E-75	2.66E-71
**Il1b**	Wound_DC_Ndrg2KO	1.50965668	4.01E-72	7.46E-68
**Il1a**	Wound_DC_Ndrg2KO	1.70326954	2.59E-71	4.82E-67
**Clic1**	Wound_DC_Ndrg2KO	0.54164103	1.39E-69	2.58E-65
**Prdx5**	Wound_DC_Ndrg2KO	0.81817749	1.01E-64	1.88E-60
**S100a8**	Wound_DC_Ndrg2KO	1.65475638	2.65E-58	4.92E-54
**Gapdh**	Wound_DC_Ndrg2KO	0.66785945	2.68E-56	4.98E-52
**Lgals1**	Wound_DC_Ndrg2KO	0.59999013	8.89E-54	1.65E-49
**Ccl4**	Wound_DC_Ndrg2KO	1.56347432	2.76E-51	5.14E-47
**Ptgs2**	Wound_DC_Ndrg2KO	1.46078691	3.36E-51	6.25E-47
**Serf2**	Wound_DC_Ndrg2KO	0.34922307	1.19E-50	2.22E-46
**Phlda1**	Wound_DC_Ndrg2KO	0.86053097	1.69E-50	3.15E-46
**Elob**	Wound_DC_Ndrg2KO	0.46319165	4.44E-50	8.27E-46
**Ccl3**	Wound_DC_Ndrg2KO	1.51017517	7.57E-50	1.41E-45
**Cxcl3**	Wound_DC_Ndrg2KO	1.87703007	3.64E-49	6.77E-45
**Tmsb4x**	Wound_DC_Ndrg2KO	0.29207725	1.81E-48	3.36E-44
**Inhba**	Wound_DC_Ndrg2KO	1.80374514	1.99E-46	3.70E-42
**Ftl1**	Wound_DC_Ndrg2KO	0.40480156	2.44E-46	4.55E-42
**S100a9**	Wound_DC_Ndrg2KO	1.51069865	5.39E-46	1.00E-41
**Upp1**	Wound_DC_Ndrg2KO	0.69572605	9.52E-44	1.77E-39
**Cyba**	Wound_DC_Ndrg2KO	0.37623318	1.86E-43	3.46E-39
**Cox8a**	Wound_DC_Ndrg2KO	0.34678304	1.97E-43	3.67E-39
**Acod1**	Wound_DC_Ndrg2KO	1.08606033	4.33E-40	8.07E-36
**Ier3**	Wound_DC_Ndrg2KO	0.52576088	4.39E-40	8.17E-36
**H3f3a**	Wound_DC_Ndrg2KO	0.31524669	6.03E-40	1.12E-35
**Tyrobp**	Wound_DC_Ndrg2KO	0.38054158	6.29E-40	1.17E-35
**Fau**	Wound_DC_Ndrg2KO	0.34287675	3.84E-39	7.16E-35
**Cox5b**	Wound_DC_Ndrg2KO	0.38144248	9.15E-39	1.70E-34
**Srgn**	Wound_DC_Ndrg2KO	0.65587152	3.06E-38	5.70E-34
**Nfkbia**	Wound_DC_Ndrg2KO	0.61212306	1.09E-37	2.03E-33
**Tnf**	Wound_DC_Ndrg2KO	1.26652971	2.61E-36	4.87E-32
**Tmsb10**	Wound_DC_Ndrg2KO	0.63466959	7.57E-36	1.41E-31
**Eif1**	Wound_DC_Ndrg2KO	0.29935231	1.53E-35	2.85E-31
**Sem1**	Wound_DC_Ndrg2KO	0.27886264	5.01E-35	9.32E-31
**Slpi**	Wound_DC_Ndrg2KO	1.22001815	1.52E-34	2.83E-30
**Ifitm3**	Wound_DC_Ndrg2KO	0.60236809	3.99E-34	7.42E-30
**Edf1**	Wound_DC_Ndrg2KO	0.38416719	4.58E-34	8.52E-30
**Txn1**	Wound_DC_Ndrg2KO	0.65101757	4.76E-34	8.85E-30
**Gng5**	Wound_DC_Ndrg2KO	0.29415208	5.81E-34	1.08E-29
**Clta**	Wound_DC_Ndrg2KO	0.41847275	1.69E-33	3.14E-29
**Nlrp3**	Wound_DC_Ndrg2KO	0.76502083	1.52E-32	2.83E-28
**Cox7c**	Wound_DC_Ndrg2KO	0.27971376	1.67E-32	3.11E-28
**Atp5h**	Wound_DC_Ndrg2KO	0.32192869	3.20E-31	5.95E-27
**Clec4e**	Wound_DC_Ndrg2KO	0.82968637	5.65E-30	1.05E-25
**Cd14**	Wound_DC_Ndrg2KO	0.53934119	7.08E-30	1.32E-25
**AA467197**	Wound_DC_Ndrg2KO	0.97508491	7.18E-30	1.34E-25
**Nfkbiz**	Wound_DC_Ndrg2KO	0.61064092	7.42E-30	1.38E-25
**Cd24a**	Wound_DC_Ndrg2KO	0.71577782	1.07E-29	2.00E-25

Table S7: Top differentially expressed genes in neutrophils of wounds treated with control dendritic cells (Wound_Controls) or Ndrg2KO dendritic cells (Wound_DC _Ndrg2KO. Differentially expressed genes were determined using a Wilcoxon Rank Sum test and P-values were adjusted using the Benjamini-Hochberg procedure as part of the Seurat package.

Gene	Group	Average log2 fold-change	P value	Adjusted p value
**Txnip**	Wound_Controls	0.8384334	1.21E-89	2.25E-85
**Mrpl52**	Wound_Controls	0.81891786	2.29E-76	4.27E-72
**Lrg1**	Wound_Controls	0.97216121	1.19E-70	2.22E-66
**Ctsd**	Wound_Controls	0.59438495	5.80E-63	1.08E-58
**Ptafr**	Wound_Controls	0.56997212	3.12E-58	5.80E-54
**Hp**	Wound_Controls	0.64359302	8.09E-57	1.51E-52
**Malat1**	Wound_Controls	0.34241373	2.30E-52	4.27E-48
**Nfam1**	Wound_Controls	0.62448536	6.31E-51	1.17E-46
**Dedd2**	Wound_Controls	0.61873369	4.51E-48	8.39E-44
**Lfng**	Wound_Controls	0.60397385	7.85E-48	1.46E-43
**Fgl2**	Wound_Controls	0.68012472	2.99E-46	5.56E-42
**F630028O10Rik**	Wound_Controls	0.69799912	1.49E-44	2.77E-40
**Csf3r**	Wound_Controls	0.47873564	8.73E-44	1.62E-39
**Asprv1**	Wound_Controls	0.65863567	2.57E-42	4.78E-38
**Igsf6**	Wound_Controls	0.58581319	4.57E-41	8.51E-37
**Ogfrl1**	Wound_Controls	0.47423111	1.25E-40	2.33E-36
**Midn**	Wound_Controls	0.48611359	5.69E-40	1.06E-35
**Lars2**	Wound_Controls	0.27692665	1.88E-39	3.50E-35
**Neat1**	Wound_Controls	0.44897273	9.28E-38	1.73E-33
**Alas1**	Wound_Controls	0.57266246	9.70E-38	1.81E-33
**Son**	Wound_Controls	0.40380689	1.28E-37	2.39E-33
**Selplg**	Wound_Controls	0.48213521	1.66E-37	3.09E-33
**Alox5ap**	Wound_Controls	0.45292187	2.56E-36	4.76E-32
**Ppia**	Wound_Controls	0.43769837	2.47E-35	4.59E-31
**2310001H17Rik**	Wound_Controls	0.47197921	3.18E-35	5.93E-31
**Fgr**	Wound_Controls	0.45038793	6.49E-35	1.21E-30
**Slc38a2**	Wound_Controls	0.39758415	6.56E-35	1.22E-30
**Zfp36l2**	Wound_Controls	0.53852346	1.08E-34	2.01E-30
**Adgre5**	Wound_Controls	0.67288216	2.70E-34	5.03E-30
**Atf7ip**	Wound_Controls	0.54678587	8.47E-34	1.58E-29
**Sirpb1b**	Wound_Controls	0.42579389	1.05E-33	1.96E-29
**Jun**	Wound_Controls	0.54210023	2.18E-33	4.06E-29
**Osgin1**	Wound_Controls	0.63313958	2.75E-33	5.11E-29
**Rnf149**	Wound_Controls	0.37879332	9.41E-33	1.75E-28
**Cd300a**	Wound_Controls	0.52045695	1.07E-32	2.00E-28
**Il1r2**	Wound_Controls	0.48191482	2.42E-32	4.50E-28
**Cxcr4**	Wound_Controls	0.67391116	2.60E-32	4.84E-28
**Marcksl1**	Wound_Controls	0.47219476	2.78E-32	5.18E-28
**Rsrp1**	Wound_Controls	0.37844026	3.23E-32	6.01E-28
**Fam111a**	Wound_Controls	0.486718	3.63E-31	6.75E-27
**Vmp1**	Wound_Controls	0.4652904	3.21E-30	5.97E-26
**Il6ra**	Wound_Controls	0.39266044	7.34E-30	1.37E-25
**BC005537**	Wound_Controls	0.41808714	1.11E-29	2.07E-25

**Gsr**	Wound_Controls	0.39227233	1.24E-29	2.30E-25
**Pdzd8**	Wound_Controls	0.408744	4.28E-29	7.97E-25
**Dhx40**	Wound_Controls	0.53715495	5.96E-29	1.11E-24
**Marcks**	Wound_Controls	0.38685147	8.73E-29	1.63E-24
**Ssh2**	Wound_Controls	0.37193239	5.48E-28	1.02E-23
**C5ar1**	Wound_Controls	0.33097356	5.80E-28	1.08E-23
**Ripor2**	Wound_Controls	0.42129331	6.70E-28	1.25E-23
**Cxcl2**	Wound_DC_Ndrg2KO	0.60745091	1.98E-60	3.68E-56
**Il1a**	Wound_DC_Ndrg2KO	1.22792606	3.36E-54	6.26E-50
**Tpt1**	Wound_DC_Ndrg2KO	0.35713971	1.19E-53	2.21E-49
**Retnlg**	Wound_DC_Ndrg2KO	0.77041426	3.17E-49	5.89E-45
**Fau**	Wound_DC_Ndrg2KO	0.32216395	5.07E-43	9.43E-39
**Prdx5**	Wound_DC_Ndrg2KO	0.51618218	5.86E-43	1.09E-38
**Slc2a3**	Wound_DC_Ndrg2KO	0.91045314	2.90E-39	5.40E-35
**Cdkn1a**	Wound_DC_Ndrg2KO	0.67174902	2.38E-36	4.43E-32
**Spp1**	Wound_DC_Ndrg2KO	0.86734911	3.70E-36	6.89E-32
**Rabgef1**	Wound_DC_Ndrg2KO	0.60319052	1.23E-35	2.29E-31
**Il1b**	Wound_DC_Ndrg2KO	0.41871247	3.21E-35	5.97E-31
**Upp1**	Wound_DC_Ndrg2KO	0.55819081	9.38E-32	1.75E-27
**Cd274**	Wound_DC_Ndrg2KO	0.75126741	1.16E-30	2.16E-26
**Tiparp**	Wound_DC_Ndrg2KO	0.58180494	4.69E-28	8.73E-24
**Basp1**	Wound_DC_Ndrg2KO	0.46852114	8.64E-27	1.61E-22
**Tmsb4x**	Wound_DC_Ndrg2KO	0.40999579	2.31E-26	4.30E-22
**Tgoln1**	Wound_DC_Ndrg2KO	0.38029961	5.24E-25	9.76E-21
**Traf1**	Wound_DC_Ndrg2KO	0.79743592	6.21E-25	1.16E-20
**Cebpb**	Wound_DC_Ndrg2KO	0.34170753	9.88E-24	1.84E-19
**Plaur**	Wound_DC_Ndrg2KO	0.50513367	1.14E-23	2.12E-19
**Csrnp1**	Wound_DC_Ndrg2KO	0.51551836	3.16E-23	5.88E-19
**Isg15**	Wound_DC_Ndrg2KO	0.71327861	1.05E-22	1.96E-18
**Vps37b**	Wound_DC_Ndrg2KO	0.59455011	4.28E-22	7.97E-18
**Ptgs2**	Wound_DC_Ndrg2KO	0.58760816	5.70E-22	1.06E-17
**Ppp1r15a**	Wound_DC_Ndrg2KO	0.43163917	5.95E-22	1.11E-17
**Hilpda**	Wound_DC_Ndrg2KO	0.75375341	2.73E-21	5.09E-17
**Ifrd1**	Wound_DC_Ndrg2KO	0.49661024	7.30E-21	1.36E-16
Nfkbiz	Wound_DC_Ndrg2KO	0.40884917	5.74E-20	1.07E-15
Ldha	Wound_DC_Ndrg2KO	0.41373075	8.75E-20	1.63E-15
Selenok	Wound_DC_Ndrg2KO	0.4071574	2.54E-19	4.73E-15
Cxcl3	Wound_DC_Ndrg2KO	0.60770394	2.85E-19	5.31E-15
Acod1	Wound_DC_Ndrg2KO	0.28331605	8.86E-19	1.65E-14
Ell2	Wound_DC_Ndrg2KO	0.41118968	2.06E-17	3.83E-13
Ccl3	Wound_DC_Ndrg2KO	0.4759164	6.85E-17	1.27E-12
Tmem33	Wound_DC_Ndrg2KO	0.45895123	1.59E-16	2.95E-12
Cyp4f18	Wound_DC_Ndrg2KO	0.36871741	3.08E-16	5.74E-12
Nampt	Wound_DC_Ndrg2KO	0.5633495	3.41E-16	6.35E-12
Rtp4	Wound_DC_Ndrg2KO	0.46926566	3.72E-16	6.92E-12
Tpi1	Wound_DC_Ndrg2KO	0.4160656	2.44E-15	4.55E-11
Rnf213	Wound_DC_Ndrg2KO	0.63328877	6.64E-15	1.24E-10
Smox	Wound_DC_Ndrg2KO	0.27295551	3.02E-14	5.63E-10
Sub1	Wound_DC_Ndrg2KO	0.40100017	4.10E-14	7.62E-10
Slc15a3	Wound_DC_Ndrg2KO	0.25904903	2.66E-13	4.95E-09
Ndel1	Wound_DC_Ndrg2KO	0.42379036	5.93E-13	1.10E-08

Clec4e	Wound_DC_Ndrg2KO	0.36196454	1.11E-12	2.07E-08
Pts	Wound_DC_Ndrg2KO	0.55757257	1.75E-12	3.26E-08
Rab20	Wound_DC_Ndrg2KO	0.26192394	1.78E-12	3.31E-08
Cd14	Wound_DC_Ndrg2KO	0.25071535	2.86E-12	5.33E-08
Dgat1	Wound_DC_Ndrg2KO	0.34782142	6.46E-12	1.20E-07
Arpc3	Wound_DC_Ndrg2KO	0.28386606	6.47E-12	1.20E-07

Table S8: Top differentially expressed genes in lymphoid cells of wounds treated with control dendritic cells (Wound_Controls) or Ndrg2KO dendritic cells (Wound_DC _Ndrg2KO. Differentially expressed genes were determined using a Wilcoxon Rank Sum test and P-values were adjusted using the Benjamini-Hochberg procedure as part of the Seurat package.

Gene	Group	Average log2 fold-change	P value	Adjusted p value
**Lars2**	Wound_Controls	0.39807811	3.62E-13	6.75E-09
**Sik1**	Wound_Controls	0.6530768	3.37E-11	6.28E-07
**Nr4a3**	Wound_Controls	0.70177356	1.13E-10	2.10E-06
**Cxcr4**	Wound_Controls	0.96969081	5.47E-10	1.02E-05
**Hnrnpa2b1**	Wound_Controls	0.49838029	3.37E-08	0.00062678
**Junb**	Wound_Controls	0.75723865	5.25E-08	0.00097752
**Vps37b**	Wound_Controls	0.76753497	6.70E-08	0.00124715
**Setd2**	Wound_Controls	0.43461982	7.80E-08	0.00145088
**Csrnp1**	Wound_Controls	0.45210524	1.37E-07	0.00255695
**Kdm6b**	Wound_Controls	0.74858098	1.51E-07	0.00280523
**Zfp36**	Wound_Controls	0.64776399	3.41E-07	0.00634271
**Impdh2**	Wound_Controls	0.4420843	3.71E-07	0.00689962
**Il4ra**	Wound_Controls	0.42853145	4.85E-07	0.00903269
**Dusp1**	Wound_Controls	0.69472386	5.68E-07	0.01057313
**Fosl2**	Wound_Controls	0.50595346	7.31E-07	0.01361036
**St13**	Wound_Controls	0.33652571	8.19E-07	0.01525285
**Fbxo11**	Wound_Controls	0.34389502	8.65E-07	0.01609691
**Tob2**	Wound_Controls	0.59441577	8.75E-07	0.01629283
**Ankrd12**	Wound_Controls	0.50378843	9.62E-07	0.01789823
**Spty2d1**	Wound_Controls	0.44200251	1.08E-06	0.02005932
**Slc38a1**	Wound_Controls	0.54212042	1.15E-06	0.02149589
**Tex2**	Wound_Controls	0.33205072	1.33E-06	0.02470105
**Huwe1**	Wound_Controls	0.4174962	1.37E-06	0.02556051
**Slc38a2**	Wound_Controls	0.65807219	1.39E-06	0.02596002
**Tuba1b**	Wound_Controls	0.33579267	1.58E-06	0.029321
**Hnrnph1**	Wound_Controls	0.4240159	1.62E-06	0.03016999
**Fosb**	Wound_Controls	0.73796615	1.79E-06	0.03339964
**Herc1**	Wound_Controls	0.35490067	2.26E-06	0.04200129
**Dennd4a**	Wound_Controls	0.56027461	3.35E-06	0.06231804
**4932438A13Rik**	Wound_Controls	0.49672755	4.86E-06	0.09053693
**Eprs**	Wound_Controls	0.57818458	5.03E-06	0.09358639
**Nup98**	Wound_Controls	0.33952624	5.59E-06	0.10413301
**Kdm2b**	Wound_Controls	0.40280621	8.27E-06	0.1539226
**Ddx5**	Wound_Controls	0.37159088	8.60E-06	0.1600053
**Ddx21**	Wound_Controls	0.42410383	9.22E-06	0.17158282
**Ern1**	Wound_Controls	0.33358784	1.33E-05	0.24704237
**Nr4a2**	Wound_Controls	0.74164556	1.37E-05	0.25548436
**Ptp4a1**	Wound_Controls	0.40661265	1.40E-05	0.26058074
**Nars**	Wound_Controls	0.40413117	1.59E-05	0.29506083
**Cebpb**	Wound_Controls	0.56500843	1.63E-05	0.30309137
**Nucks1**	Wound_Controls	0.34334541	1.75E-05	0.32613531
**Smg7**	Wound_Controls	0.27493659	1.85E-05	0.34391573
**Clk1**	Wound_Controls	0.46225826	1.95E-05	0.36237982

**Tgif1**	Wound_Controls	0.54158472	2.08E-05	0.38793867
**Ccnl1**	Wound_Controls	0.32682948	2.42E-05	0.45119119
**Atp1a1**	Wound_Controls	0.35104045	2.45E-05	0.45589449
**Mylip**	Wound_Controls	0.31495987	2.55E-05	0.47422193
**Ctnnb1**	Wound_Controls	0.33204848	2.96E-05	0.55143335
**Nr4a1**	Wound_Controls	0.58119977	3.10E-05	0.5761575
**Kcnq1ot1**	Wound_Controls	0.53614858	3.40E-05	0.63299795
**Fau**	Wound_DC_Ndrg2KO	0.47570825	3.13E-22	5.83E-18
**Cd52**	Wound_DC_Ndrg2KO	0.76265876	8.96E-14	1.67E-09
**Rgs10**	Wound_DC_Ndrg2KO	0.98400226	5.54E-12	1.03E-07
**Ifitm2**	Wound_DC_Ndrg2KO	1.41339484	9.14E-12	1.70E-07
**Ptpn18**	Wound_DC_Ndrg2KO	0.55735636	1.27E-10	2.36E-06
**Cxcl2**	Wound_DC_Ndrg2KO	0.51425004	3.98E-10	7.40E-06
**Sh3bgrl3**	Wound_DC_Ndrg2KO	0.59545612	9.98E-10	1.86E-05
**Uqcrh**	Wound_DC_Ndrg2KO	0.48701494	4.25E-09	7.92E-05
**Cox8a**	Wound_DC_Ndrg2KO	0.45416704	4.26E-09	7.94E-05
**Psmb8**	Wound_DC_Ndrg2KO	0.58772954	6.57E-09	0.00012222
**S100a13**	Wound_DC_Ndrg2KO	0.55441294	7.25E-09	0.00013495
**Cd47**	Wound_DC_Ndrg2KO	0.55939717	2.76E-08	0.00051449
**Ifitm3**	Wound_DC_Ndrg2KO	1.05353296	2.93E-08	0.0005458
**Tmsb4x**	Wound_DC_Ndrg2KO	0.44288373	4.36E-08	0.00081178
**Ms4a6b**	Wound_DC_Ndrg2KO	0.79253287	4.74E-08	0.00088289
**Dnajc15**	Wound_DC_Ndrg2KO	0.63723055	5.38E-08	0.001001
**Ifitm1**	Wound_DC_Ndrg2KO	2.05387089	7.71E-08	0.00143529
**S100a10**	Wound_DC_Ndrg2KO	0.54379926	8.06E-08	0.00150047
**Gng2**	Wound_DC_Ndrg2KO	0.59667994	8.89E-08	0.00165539
**S100a6**	Wound_DC_Ndrg2KO	0.61233659	1.42E-07	0.00264145
**Clic1**	Wound_DC_Ndrg2KO	0.49315556	1.59E-07	0.00295858
**Arhgdib**	Wound_DC_Ndrg2KO	0.50305614	1.60E-07	0.0029752
**Pfdn5**	Wound_DC_Ndrg2KO	0.44821228	1.73E-07	0.00322792
**Ms4a4b**	Wound_DC_Ndrg2KO	0.74186573	1.82E-07	0.00339503
**Cd3d**	Wound_DC_Ndrg2KO	0.60144493	4.69E-07	0.00872384
**Lgals1**	Wound_DC_Ndrg2KO	0.65704575	7.89E-07	0.01468462
**AW112010**	Wound_DC_Ndrg2KO	0.74143443	8.33E-07	0.01549735
**Cox4i1**	Wound_DC_Ndrg2KO	0.3779902	9.73E-07	0.0181119
**Ly6a**	Wound_DC_Ndrg2KO	0.75662768	1.10E-06	0.02044072
**Rac2**	Wound_DC_Ndrg2KO	0.4184309	2.11E-06	0.03924078
**Coro1a**	Wound_DC_Ndrg2KO	0.40211254	2.33E-06	0.04329981
**Fxyd5**	Wound_DC_Ndrg2KO	0.47811114	2.86E-06	0.05329632
**Trbc2**	Wound_DC_Ndrg2KO	0.68193121	4.30E-06	0.08009498
**Cd3g**	Wound_DC_Ndrg2KO	0.57066166	6.55E-06	0.12187152
**Slfn1**	Wound_DC_Ndrg2KO	0.57075621	7.74E-06	0.14408454
**Ctla2a**	Wound_DC_Ndrg2KO	0.62232569	8.44E-06	0.15716759
**Tspo**	Wound_DC_Ndrg2KO	0.42553807	1.19E-05	0.22152818
**Lgals3**	Wound_DC_Ndrg2KO	1.05330249	1.46E-05	0.27238827
**Elob**	Wound_DC_Ndrg2KO	0.37171602	2.43E-05	0.45244481
**Mif**	Wound_DC_Ndrg2KO	0.54968953	3.00E-05	0.55899564
**Cox6b1**	Wound_DC_Ndrg2KO	0.3577062	3.20E-05	0.59632482
**Slfn2**	Wound_DC_Ndrg2KO	0.52292516	3.73E-05	0.6938769
**Itgb1**	Wound_DC_Ndrg2KO	0.48965831	4.08E-05	0.75887475

**Arf5**	Wound_DC_Ndrg2KO	0.43261204	5.97E-05	1
**AA467197**	Wound_DC_Ndrg2KO	0.40676839	6.78E-05	1
**Aldoa**	Wound_DC_Ndrg2KO	0.61616468	7.20E-05	1
**H2-K1**	Wound_DC_Ndrg2KO	0.27309117	0.00010017	1
**Laptm5**	Wound_DC_Ndrg2KO	0.35115269	0.00010218	1
**Myl6**	Wound_DC_Ndrg2KO	0.35653691	0.00010859	1
**Naca**	Wound_DC_Ndrg2KO	0.27118392	0.0001088	1

Supplementary Figure 1

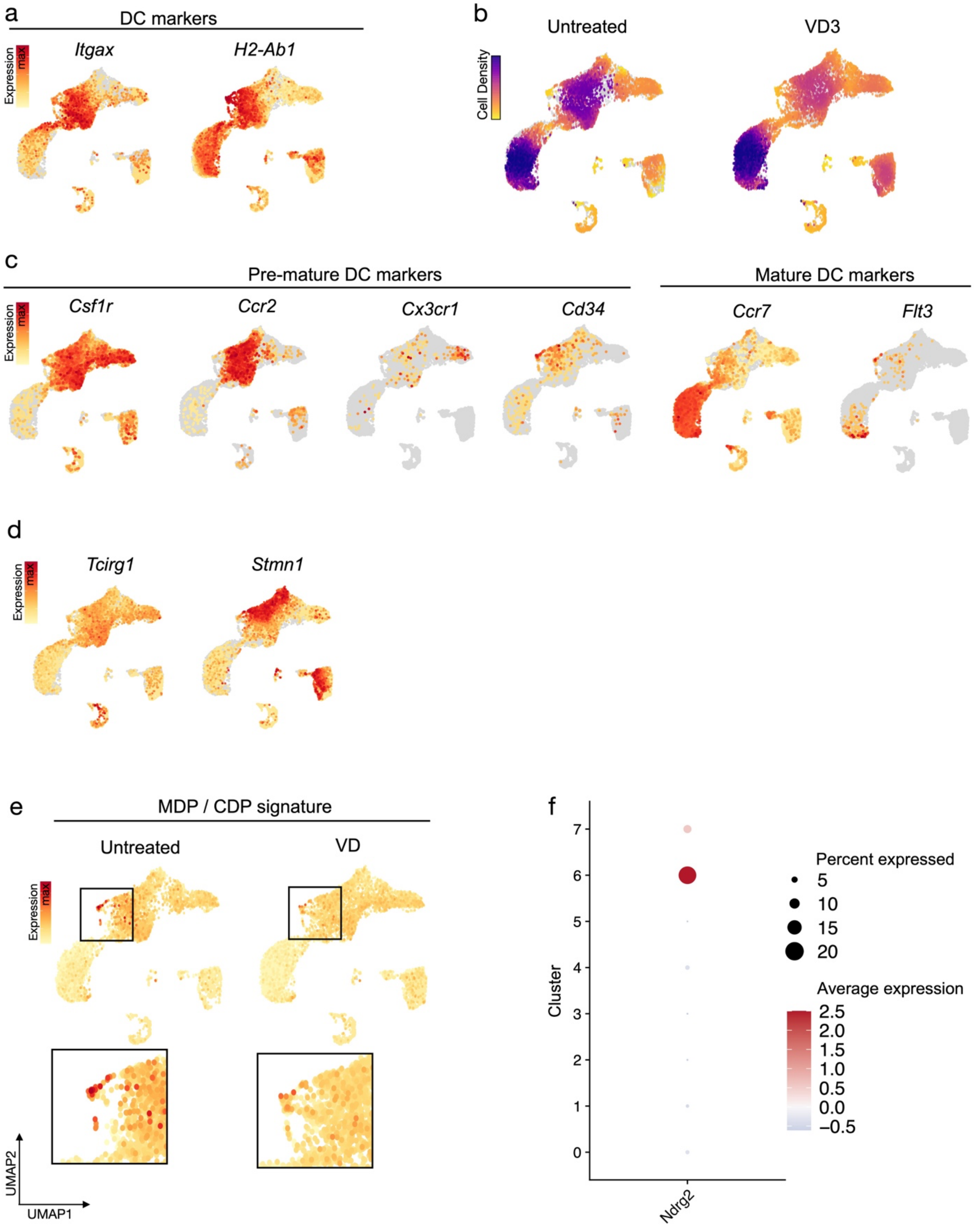

Supplementary Figure 1

a, Expression of *Itagx* and *H2-Ab1* projected onto UMAP embedding of Figure 1. **b**, UMAP embedding of Fig. 1 with untreated and vitamin D3 (VD3) treated cells colored by cell density. **c,** Expression of markers pre-mature and mature DCs projected onto the UMAP embedding. **d,** Expression of *Tcirg1* and *Stmn1* projected onto UMAP embedding. **e**, macrophage dendritic cell progenitor (MDP) / common dendritic cell progenitor (CDP) expression score computed using expression levels for *Ndrg2, Csf1r, Flt3, Clec9a* projected onto UMAP embedding. **f**, Dot plot indicating *Ndrg2* expression in different Seurat clusters of Figure 1b.

Supplementary Figure 2

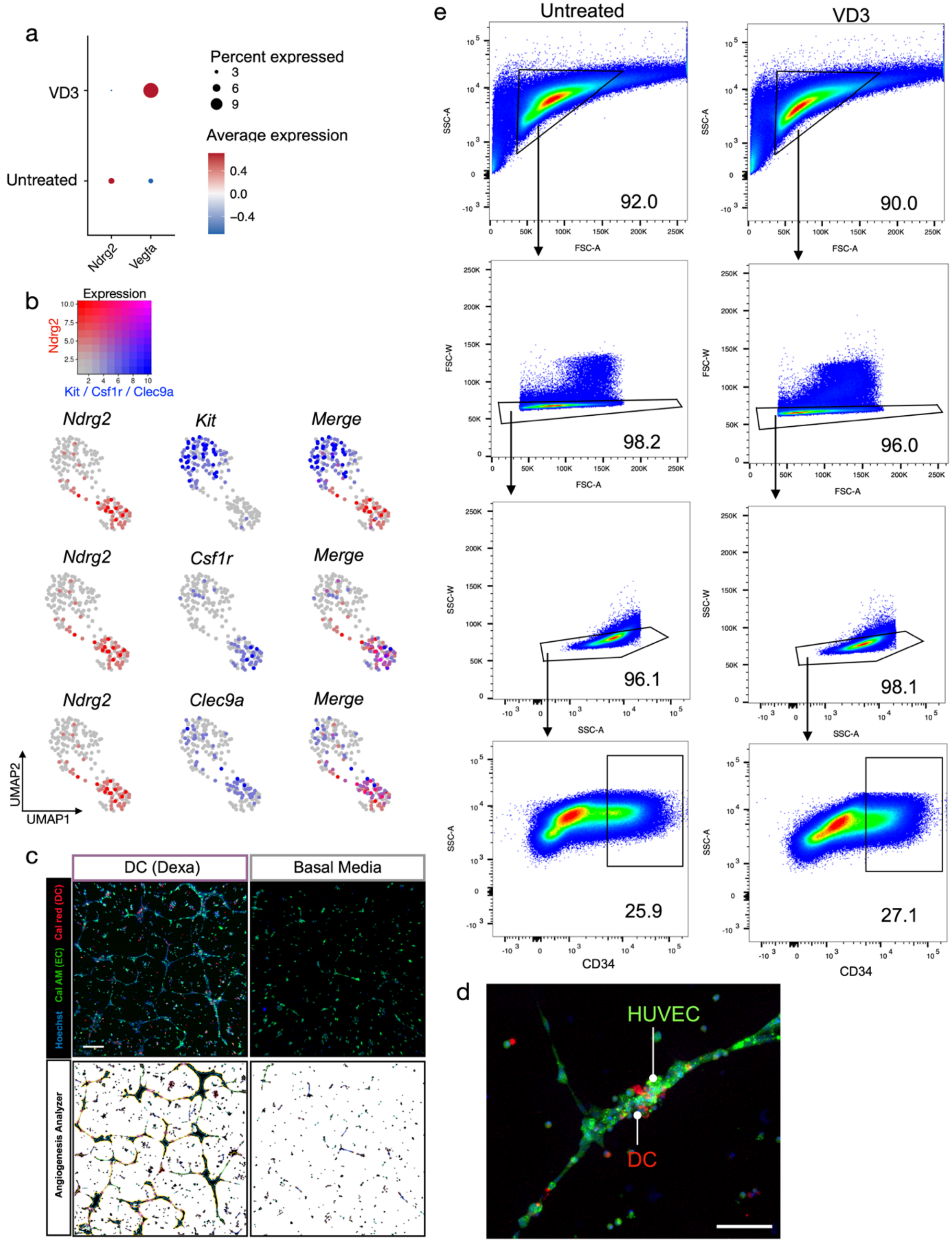

Supplementary Figure 2

a, Dot plot indicating *Ndrg2* and *Vegfa* expression in vitamin D3 (VD3) treated and control (untreated) cells. **b**, Expression of *Ndrg2* (red) and *Kit*, *Csf1r*, and *Clec9a* (blue) projected onto UMAP embedding of cluster 6 subset. Right column shows merged expression of *Ndrg2* and co-expressed genes. **c**, Endothelial cell (EC) tube formation after co-culture of ECs together with dexamethasone stimulated dendritic cells (DCs) and after EC monoculture in basal media (negative control), n = 3 biological replicates. **d**, Magnified image of EC tube formation after co-culture of ECs with VD3-stimulated DCs indicating close interaction of DCs and ECs. Scale bars: 200 µm in overview and 100 µm in magnified image. **e**, Pre-gating strategy for untreated and VD3 treated DCs in Figure 1f.

Supplementary Figure 3

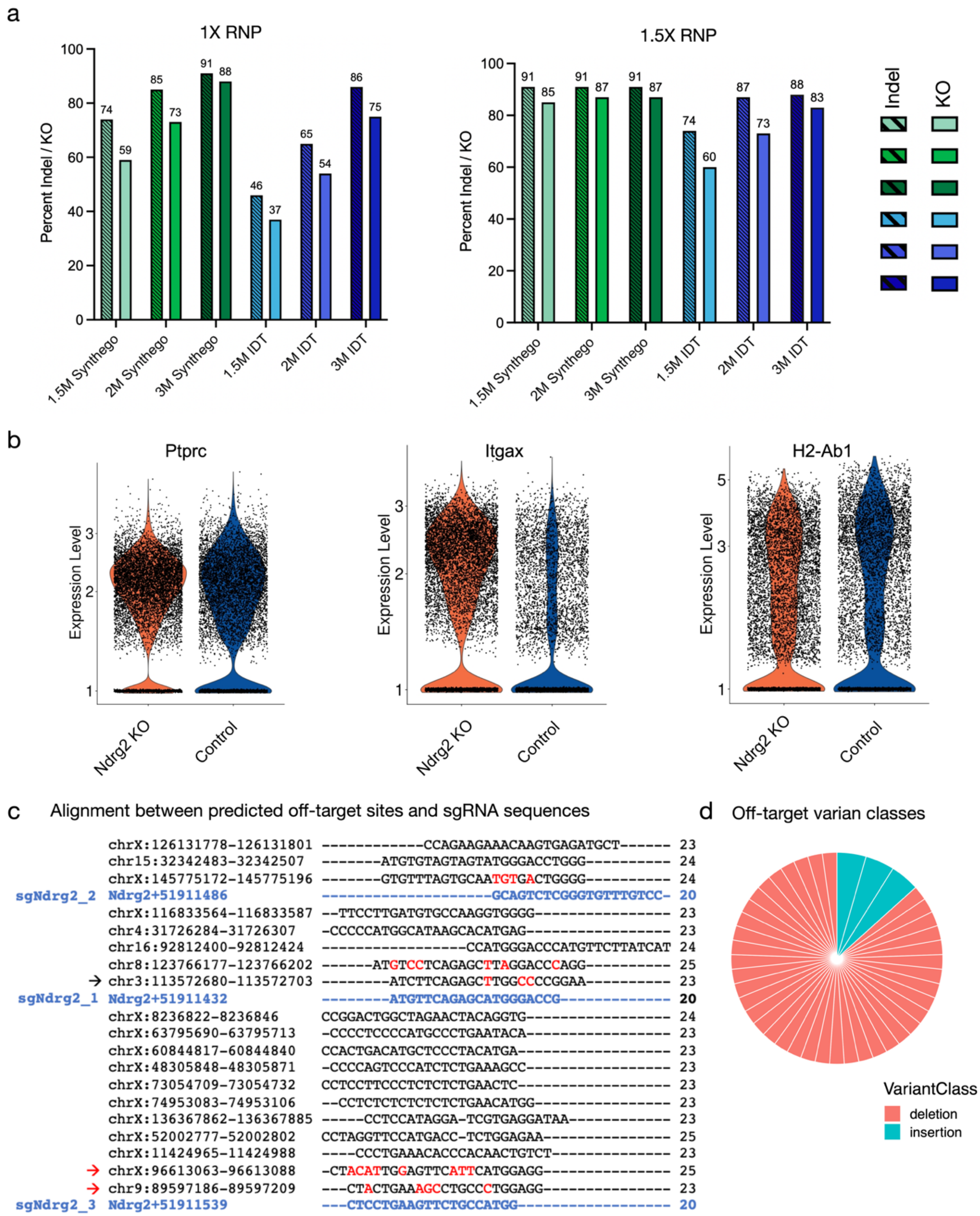

Supplementary Figure 3

a, Optimization of Cas9 RNP approach using sgRNAs obtained from different manufacturers (Synthego and Integrated DNA Technologies, IDT) at different cell concentrations (1.5 M, 2 M, 3 M). Left: Nucleofection performed with 18.6 pmol Cas9 and 22 pmol sgRNA mix (1 X RNP), right: Nucleofection performed with 27.9 pmol Cas9 and 33 pmol sgRNA mix (1.5 X RNP). **b**, single-cell RNA sequencing of Ndrg2-knockout (Ndrg2-KO) and control (untreated) dendritic cells. The expression of *Ptprc*, *Itgax*, and *H2-Ab1* is shown as violin plots. **c**, Alignment between predicted off-target sites and sgRNA sequences (blue). Rows with arrows demonstrate a high alignment. Red arrows indicate potential off-target sites that show a protospacer adjacent motif (PAM) sequence. **d**, Off-target variant classes. White lines indicate the numbers of different consequences per group.

Supplementary Figure 4

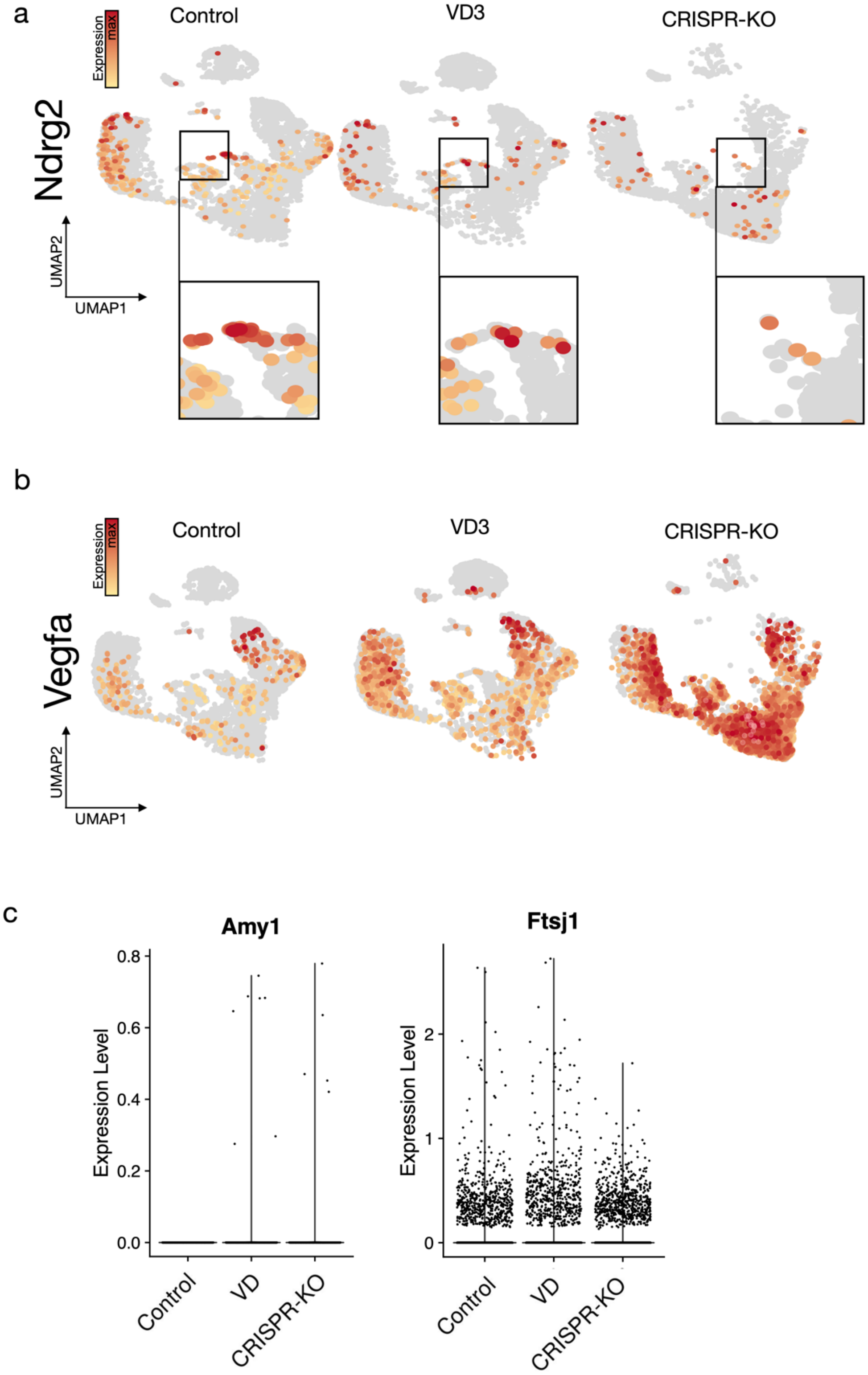

Supplementary Figure 4

a, *Ndrg2* expression projected onto UMAP embedding of in vitro scRNA-seq dataset, plot split by experimental group. Zoom-in panels showing cluster 9 (Ndrg2+ progenitors). **b**, *Vegfa* expression projected onto UMAP embedding of in vitro scRNA-seq dataset, plot split by experimental group. **c**, Violin plots showing expression of genes (*Amy1* and *Ftsj1*) at potential off-target sites.

Supplementary Figure 5

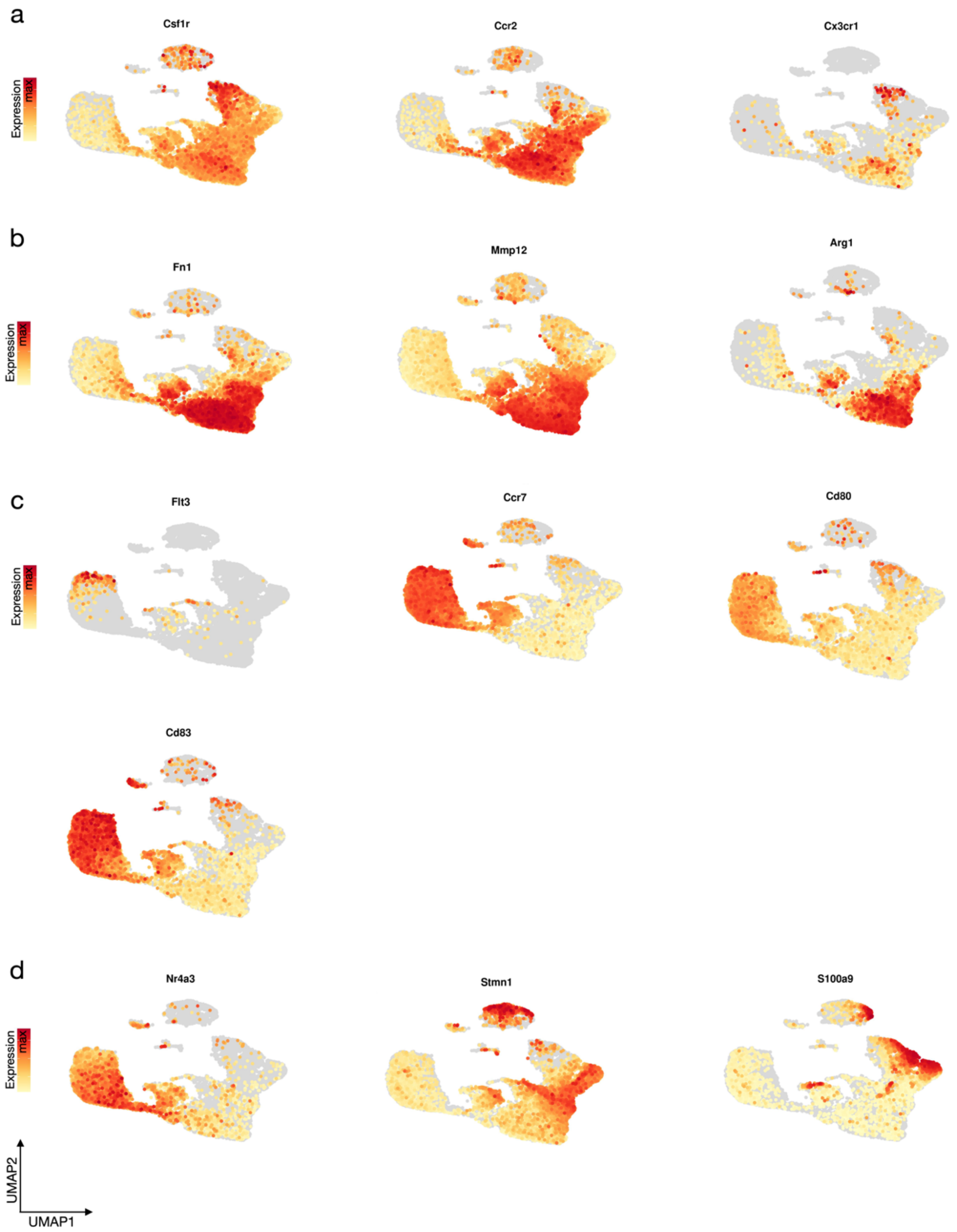

Supplementary Figure 5

a, Expression of markers of premature dendritic cells (DCs) projected onto UMAP embedding of Figure 3. **b,** Expression of *Fn1, Mmp12, Arg1* projected onto UMAP embedding. **c,** Expression of mature DC markers projected onto UMAP embedding. **d,** Expression of *Nr4a3, Stmn1, S100a9* projected onto UMAP embedding.

Supplementary Figure 6

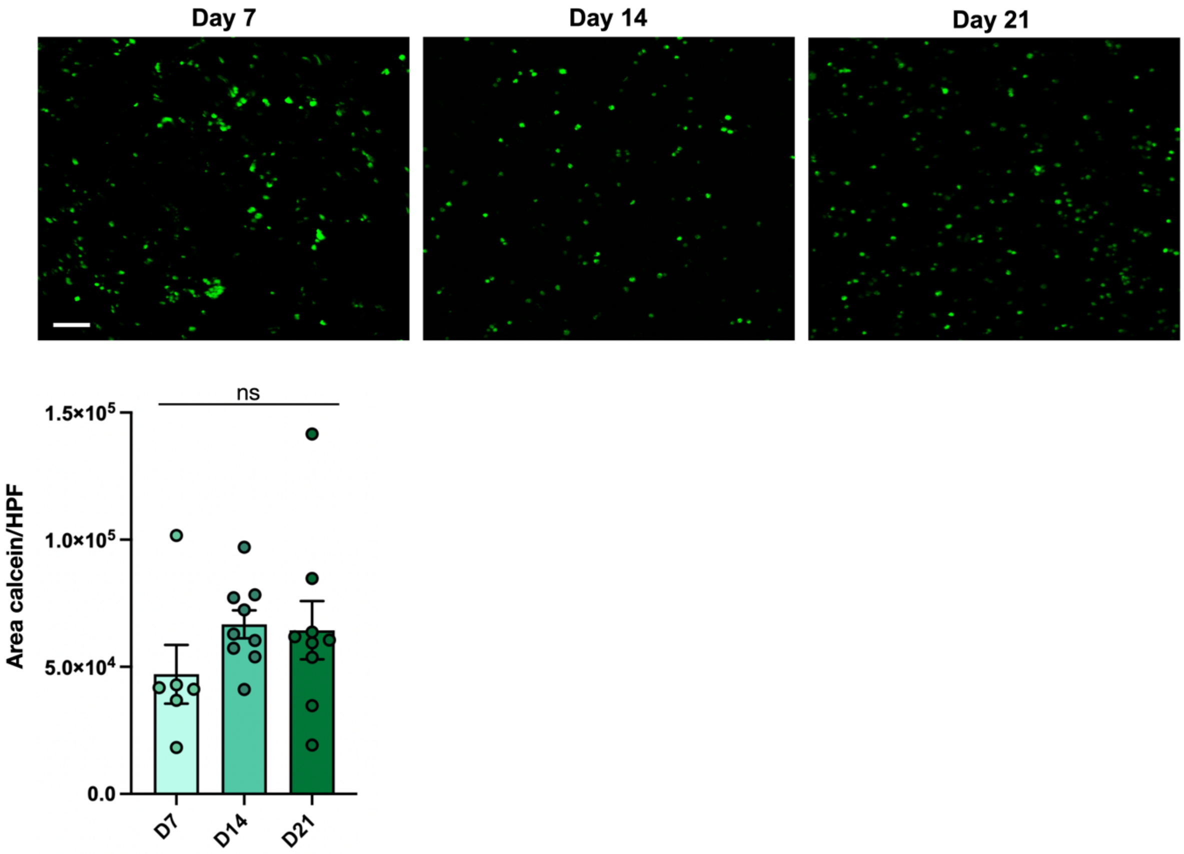

Supplementary Figure 6

Dendritic cells cultured on collagen-pullulan hydrogels for 7, 14, and 21 days labeled with Calcein AM (green). Scale bar: 200 μm. The bar plot shows the quantification of calcein AM (green) area per high power field (HPF), n = 8 biological replicates for Day 7, n = 9 biological replicates for D14, n = 9 biological replicates for Day 21. One-way analysis of variance (ANOVA) with Tukey's multiple comparisons test. P = 0.36. Data are presented as mean values +/- SEM.

Supplementary Figure 7

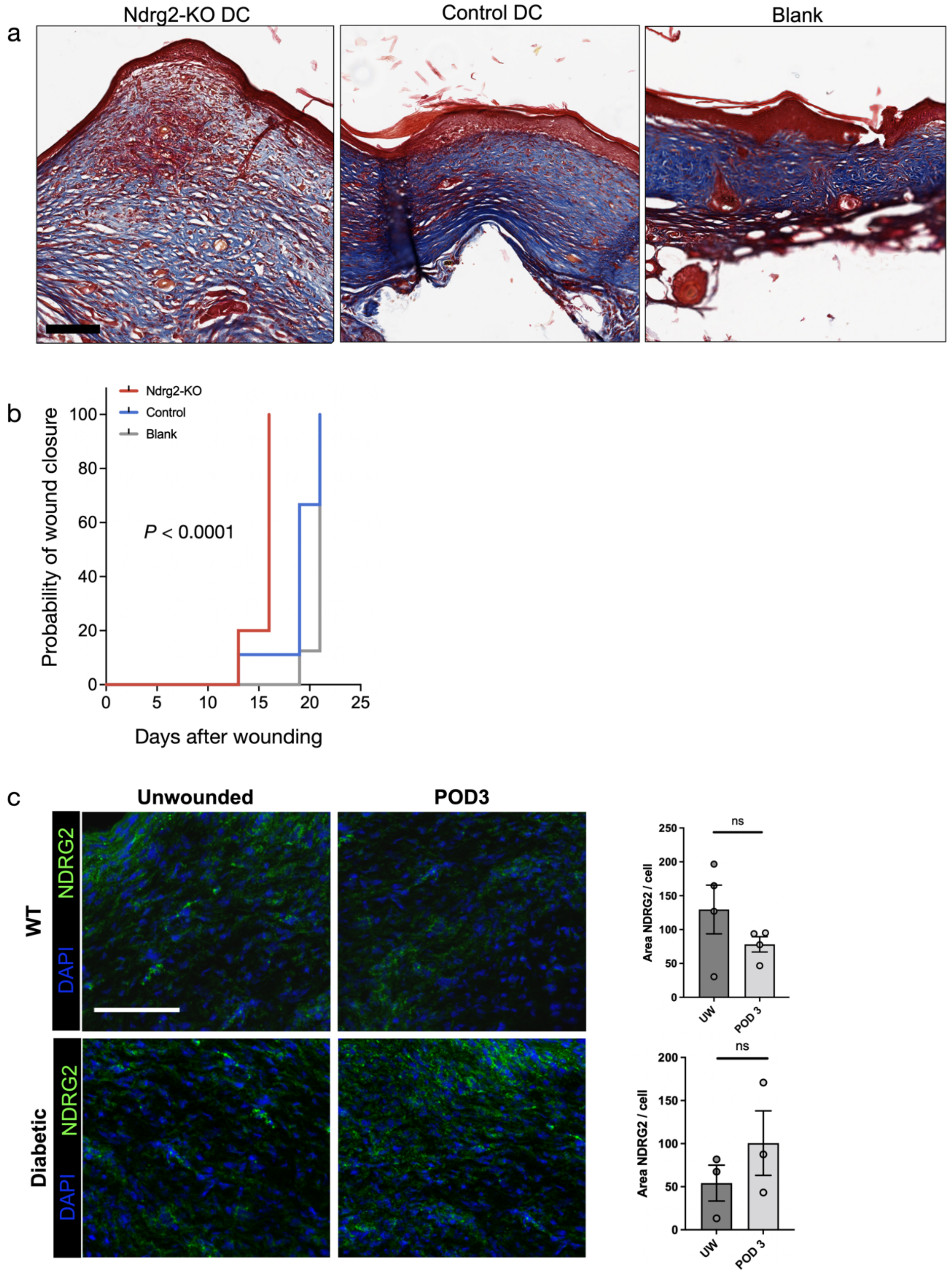

Supplementary Figure 7

a, Masson's trichrome staining of explanted wound tissue from diabetic mice (day 21). Scale bar: 200 µm. **b**, Probability of wound closure in diabetic mice after treatment with Ndrg2-KO dendritic cells (DCs), control DCs (nucleofection with 3 non-targeting sgRNAs), and blank hydrogels (reverse Kaplan-Meier estimate, ****P < 0.0001), n = 7 biological replicates for Ndrg2-KO, n = 4 biological replicates for Control, n = 7 biological replicates for Blank. **c**, Immunofluorescent staining of unwounded skin and wound tissue explanted on postoperative day (POD) 3 for NDRG2 in wild-type (WT, n = 4 biological replicates per group) and diabetic (db/db) mice (n = 3 biological replicates per group). Unpaired two-tailed T-test: P = 0.22 for WT and 0.34 for db/db mice. Data are presented as mean values +/- SEM. Scale bar: 200 µm.

Supplementary Figure 8

a

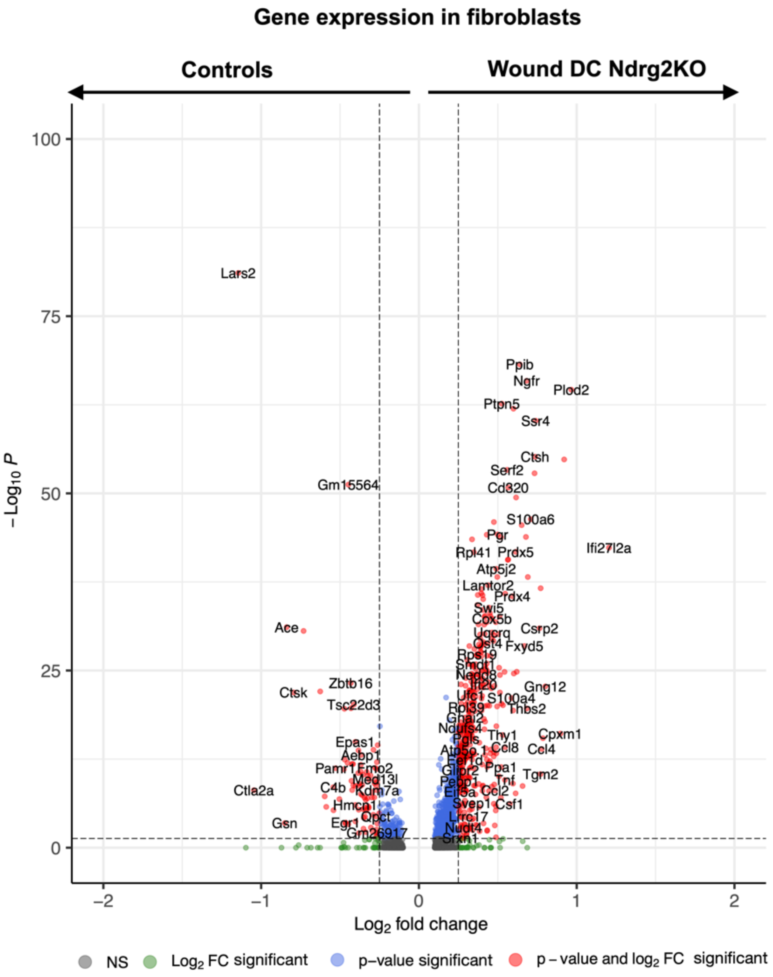

b

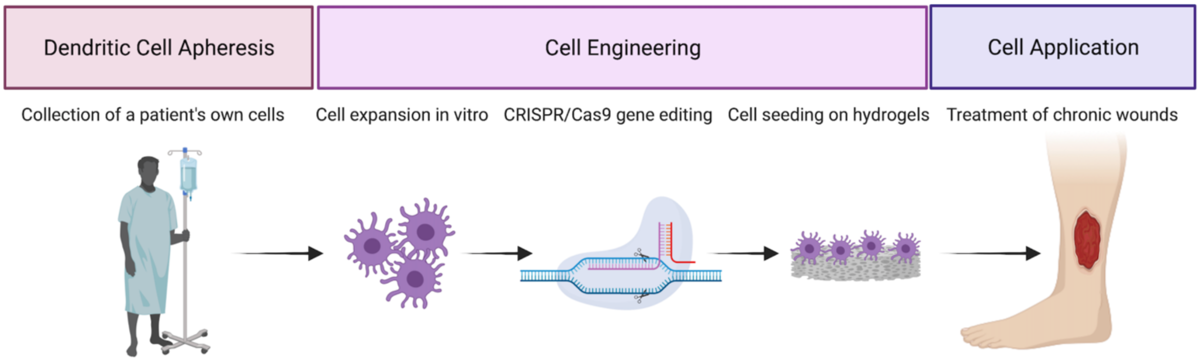

Supplementary Figure 8

a, Volcano plot showing gene expression in fibroblasts from excisional wounds that had been treated with Ndrg2KO dendritic cells (DCs) or control DCs (controls). Differentially expressed genes were determined using a Wilcoxon Rank Sum test and P-values were adjusted using the Benjamini-Hochberg procedure as part of the Seurat package. **b**, Workflow for possible future clinical application of genetically edited DCs for the treatment of chronic wounds.
