# Supplementary material for: Cas9-mediated knockout of Ndrg2 enhances the regenerative potential of dendritic cells for wound healing
Source: Nat Commun. 2023 Aug 7;14:4729. doi: 10.1038/s41467-023-40519-z (PMC10406832; doi:10.1038/s41467-023-40519-z)
Supplement: Supplementary file 3 — Reporting Summary [file 41467_2023_40519_MOESM3_ESM.pdf]

nature portfolio

Corresponding author(s): Lei S. Qi, Geoffrey C. Gurtner, Kellen Chen

Last updated by author(s): Feb 13, 2023

Reporting Summary

Nature Portfolio wishes to improve the reproducibility of the work that we publish. This form provides structure for consistency and transparency in reporting. For further information on Nature Portfolio policies, see our Editorial Policies and the Editorial Policy Checklist.

Statistics

For all statistical analyses, confirm that the following items are present in the figure legend, table legend, main text, or Methods section.

n/a	Confirmed	
☐	☒	The exact sample size (*n*) for each experimental group/condition, given as a discrete number and unit of measurement
☐	☒	A statement on whether measurements were taken from distinct samples or whether the same sample was measured repeatedly
☐	☒	The statistical test(s) used AND whether they are one- or two-sided<br>*Only common tests should be described solely by name; describe more complex techniques in the Methods section.*
☒	☐	A description of all covariates tested
☐	☒	A description of any assumptions or corrections, such as tests of normality and adjustment for multiple comparisons
☐	☒	A full description of the statistical parameters including central tendency (e.g. means) or other basic estimates (e.g. regression coefficient) AND variation (e.g. standard deviation) or associated estimates of uncertainty (e.g. confidence intervals)
☐	☒	For null hypothesis testing, the test statistic (e.g. *F*, *t*, *r*) with confidence intervals, effect sizes, degrees of freedom and *P* value noted<br>*Give P values as exact values whenever suitable.*
☒	☐	For Bayesian analysis, information on the choice of priors and Markov chain Monte Carlo settings
☒	☐	For hierarchical and complex designs, identification of the appropriate level for tests and full reporting of outcomes
☒	☐	Estimates of effect sizes (e.g. Cohen's *d*, Pearson's *r*), indicating how they were calculated

Our web collection on statistics for biologists contains articles on many of the points above.

Software and code

Policy information about availability of computer code

Data collection	No software was used for data collection.
Data analysis	R packages Seurat (version 3.1.1), scVelo (v0.2.3), GeneTrail (v3.0), SingleR (v3.11), CytoTRACE(v1, code from https:// cytotrace.stanford.edu) were used for single cell analyses. MatFiber code was previously developed and may be found on GitHub at https:// github.com/ cardiacbiomech anicsgroup/MatFiber. Prism8 was used for statistical analysis.

For manuscripts utilizing custom algorithms or software that are central to the research but not yet described in published literature, software must be made available to editors and reviewers. We strongly encourage code deposition in a community repository (e.g. GitHub). See the Nature Portfolio guidelines for submitting code & software for further information.

Data

Policy information about availability of data

All manuscripts must include a data availability statement. This statement should provide the following information, where applicable:

- Accession codes, unique identifiers, or web links for publicly available datasets
- A description of any restrictions on data availability
- For clinical datasets or third party data, please ensure that the statement adheres to our policy

Sequencing reads were aligned to the mouse reference genome (GRCm38). The authors declare that the source data supporting the findings of this study are provided with the manuscript and supplementary information files. The scRNA-seq data discussed in this publication have been deposited in NCBI's Gene Expression

Omnibus and are accessible through GEO Series accession number . Automated cell- level annotations were ascribed using the SingleR toolkit (version 3.11) against the ImmGen database.

Human research participants

Policy information about studies involving human research participants and Sex and Gender in Research.

Reporting on sex and gender	NA
Population characteristics	NA
Recruitment	NA
Ethics oversight	NA

Note that full information on the approval of the study protocol must also be provided in the manuscript.

Field-specific reporting

Please select the one below that is the best fit for your research. If you are not sure, read the appropriate sections before making your selection.

☒ Life sciences ☐ Behavioural & social sciences ☐ Ecological, evolutionary & environmental sciences

For a reference copy of the document with all sections, see nature.com/documents/nr-reporting-summary-flat.pdf

Life sciences study design

All studies must disclose on these points even when the disclosure is negative.

Sample size	The mice were assigned randomly to the different treatment groups (n = 5 mice per condition) to receive either Ndrg2-KO cells, control DCs or blank hydrogels. A sample size of n=5 mice per group is common for highly inbred mouse strains and was determined according to previously published studies using the stented full-thickness excisional wound model.
Data exclusions	No data was excluded.
Replication	To verify the reproducibility of our findings, we performed each major experiment multiple times in order to generate biological replicates. All attempts at replication were successful. For mouse studies, 5 mice were used per treatment condition. All attempts at replication of the mouse experiments were successful. For in vitro tube formation assays 3 independent replicates (cell culture assays) were used. For staining of cultured dendritic cells, 6 independent replicates were used.
Randomization	The mice were randomly assigned to receive either Ndrg2-KO cells, control DCs or blank hydrogels. Cell culture plates of dendritic cells were randomly assigned to undergo knockout of Ndrg2 using Cas9-RNP or remain untreated.
Blinding	Days to wound closure were determined for each wound based on blinded assessment of gross photography. Immunofluorescent staining was made in an unbiased manner using MATLAB image processing to quantify the amount of stain in each sample. The same threshold was used for all images. For scRNA-seq, cells were processed by a core facility (SFGF) blinded to treatment allocation. For analysis, data were normalized and processed according to the Seurat package in an unbiased manner. The investigators were blinded to group allocation during data collection and analysis.

Behavioural & social sciences study design

All studies must disclose on these points even when the disclosure is negative.

Study description	NA
Research sample	NA
Sampling strategy	NA
Data collection	NA
Timing	NA
Data exclusions	NA

Non-participation	NA
Randomization	NA

Ecological, evolutionary & environmental sciences study design

All studies must disclose on these points even when the disclosure is negative.

Study description	NA
Research sample	NA
Sampling strategy	NA
Data collection	NA
Timing and spatial scale	NA
Data exclusions	NA
Reproducibility	NA
Randomization	NA
Blinding	NA

Did the study involve field work? ☐ Yes ☒ No

Reporting for specific materials, systems and methods

We require information from authors about some types of materials, experimental systems and methods used in many studies. Here, indicate whether each material, system or method listed is relevant to your study. If you are not sure if a list item applies to your research, read the appropriate section before selecting a response.

Materials & experimental systems

n/a	Involved in the study	
☐	☒	Antibodies
☒	☐	Eukaryotic cell lines
☒	☐	Palaeontology and archaeology
☐	☒	Animals and other organisms
☒	☐	Clinical data
☒	☐	Dual use research of concern

Methods

n/a	Involved in the study	
☒	☐	ChIP-seq
☐	☒	Flow cytometry
☒	☐	MRI-based neuroimaging

Antibodies

Antibodies used	Immunofluorescent staining was performed using primary antibodies targeting CD31 (ab28364; Abcam, Waltham, MA) and Ndrg2 (ab174850; Abcam, Waltham, MA).
Validation	All antibodies used were validated by the suppliers they were acquired from and were used at the optimized conditions according to the manufacturer's recommendations. Additional validation information and relevant publications are available on the manufacturers' website: https://www.abcam.com/primary-antibodies/how-we-validate-our-antibodies

Eukaryotic cell lines

Policy information about cell lines and Sex and Gender in Research

Cell line source(s)	No cell lines were used.
Authentication	NA

Mycoplasma contamination	NA
Commonly misidentified lines (See ICLAC register)	NA

Palaeontology and Archaeology

Specimen provenance	NA
Specimen deposition	NA
Dating methods	NA

☐ Tick this box to confirm that the raw and calibrated dates are available in the paper or in Supplementary Information.

Ethics oversight	NA

Note that full information on the approval of the study protocol must also be provided in the manuscript.

Animals and other research organisms

Policy information about studies involving animals; ARRIVE guidelines recommended for reporting animal research, and Sex and Gender in Research

Laboratory animals	C57BL/6J (wild-type) and C57/BL/6-db/db mice were obtained from the Jackson Laboratory (Bar Harbor, ME). Only 6 weeks old mice were used for experiments. Animals were housed under standard conditions according to protocols of the Stanford University Institutional Animal Care and Use Committees in a 24-hour dark/light cycle and a temperature and humidity-controlled environment with access to food and water ad libitum.
Wild animals	The study did not involve wild animals.
Reporting on sex	Female and male mice were randomly assigned to the different treatment groups to receive wound treatment with either Ndrg2-KO cells, control DCs or blank hydrogels.
Field-collected samples	The study did not involve samples collected from the field.
Ethics oversight	All experiments were performed in accordance with Stanford University Institutional Animal Care and Use Committees and the NIH Guide for the Care and Use of Laboratory Animals. The study was approved by the Administrative Panel on Laboratory Animal Care (APLAC) at Stanford University (APLAC protocol number: APB-2965-GG0619).

Note that full information on the approval of the study protocol must also be provided in the manuscript.

Clinical data

Policy information about clinical studies

All manuscripts should comply with the ICMJE guidelines for publication of clinical research and a completed CONSORT checklist must be included with all submissions.

Clinical trial registration	NA
Study protocol	NA
Data collection	NA
Outcomes	NA

Dual use research of concern

Policy information about dual use research of concern

Hazards

Could the accidental, deliberate or reckless misuse of agents or technologies generated in the work, or the application of information presented in the manuscript, pose a threat to:

No	Yes	
☒	☐	Public health
☒	☐	National security
☒	☐	Crops and/or livestock
☒	☐	Ecosystems
☒	☐	Any other significant area

Experiments of concern

Does the work involve any of these experiments of concern:

No	Yes	
☒	☐	Demonstrate how to render a vaccine ineffective
☒	☐	Confer resistance to therapeutically useful antibiotics or antiviral agents
☒	☐	Enhance the virulence of a pathogen or render a nonpathogen virulent
☒	☐	Increase transmissibility of a pathogen
☒	☐	Alter the host range of a pathogen
☒	☐	Enable evasion of diagnostic/detection modalities
☒	☐	Enable the weaponization of a biological agent or toxin
☒	☐	Any other potentially harmful combination of experiments and agents

ChIP-seq

Data deposition

☐ Confirm that both raw and final processed data have been deposited in a public database such as GEO.

☐ Confirm that you have deposited or provided access to graph files (e.g. BED files) for the called peaks.

Data access links<br>*May remain private before publication.*	ChIP seq was not used in this study.
Files in database submission	NA
Genome browser session<br>(e.g. UCSC)	NA

Methodology

Replicates	NA
Sequencing depth	NA
Antibodies	NA
Peak calling parameters	NA
Data quality	NA
Software	NA

Flow Cytometry

Plots

Confirm that:

☒ The axis labels state the marker and fluorochrome used (e.g. CD4-FITC).

☒ The axis scales are clearly visible. Include numbers along axes only for bottom left plot of group (a 'group' is an analysis of identical markers).

☒ All plots are contour plots with outliers or pseudocolor plots.

☒ A numerical value for number of cells or percentage (with statistics) is provided.

Methodology

Sample preparation	Bone marrow derived dendritic cells iwere washed in FACS buffer and stained with PE anti-mouse CD135 antibody, Brilliant Violet 421 anti-mouse CD103 antibody, and Alexa Fluor® 647 anti-mouse CD34 antibody according to the manufacturer's recommendation (Biolegend, San Diego, CA).
Instrument	BD FACS Aria (Becton Dickinson, San Jose, CA) at the Stanford University Flow cytometry core facility.
Software	FlowJo (Becton Dickinson, San Jose, CA) provided by the Stanford University Flow cytometry core facility.
Cell population abundance	Flow cytometry showed that 3% of all CD34+ DC progenitors in untreated cultures co-expressed Flt3 (CD135) and CD103, while this population was reduced to 0.8% after VD3 treatment.
Gating strategy	Preliminary gating strategy consisted of FSC-A/SSC-A, FSC-A/FSC-W, and SSC-A/SSC-W gates to identify single cells and exclude doublets.

☒ Tick this box to confirm that a figure exemplifying the gating strategy is provided in the Supplementary Information.

Magnetic resonance imaging

Experimental design

Design type	Magnetic resonance imaging was not used in this study.
Design specifications	NA
Behavioral performance measures	NA

Acquisition

Imaging type(s)	NA
Field strength	NA
Sequence & imaging parameters	NA
Area of acquisition	NA

Diffusion MRI ☐ Used ☒ Not used

Preprocessing

Preprocessing software	NA
Normalization	NA
Normalization template	NA
Noise and artifact removal	NA
Volume censoring	NA

Statistical modeling & inference

Model type and settings	NA
Effect(s) tested	NA

Specify type of analysis: ☐ Whole brain ☐ ROI-based ☐ Both

Statistic type for inference (See Eklund et al. 2016)	NA
Correction	NA

Models & analysis

n/a	Involved in the study
☒	☐ Functional and/or effective connectivity
☒	☐ Graph analysis
☒	☐ Multivariate modeling or predictive analysis
